# ByteTrack: a deep learning approach for bite count and bite rate detection using meal videos in children

**DOI:** 10.3389/fnut.2025.1610363

**Published:** 2025-10-03

**Authors:** Yashaswini Rajendra Bhat, Kathleen L. Keller, Timothy R. Brick, Alaina L. Pearce

**Affiliations:** ^1^Department of Nutritional Sciences, Pennsylvania State University, University Park, PA, United States; ^2^Department of Food Science, Pennsylvania State University, University Park, PA, United States; ^3^Department of Human Development and Family Studies, Pennsylvania State University, University Park, PA, United States; ^4^Institute of Computational and Data Sciences, Pennsylvania State University, University Park, PA, United States

**Keywords:** bite detection, neural networks, eating behaviors, childhood obesity, dietary assessment, automation

## Abstract

**Introduction:**

Assessing eating behaviors such as eating rate can shed light on risk for overconsumption and obesity. Current approaches either use sensors that disrupt natural eating or rely on labor-intensive video coding, which limits scalability.

**Methods:**

We developed ByteTrack, a deep learning system for automated bite count and bite-rate detection from video-recorded child meals. The dataset comprised 1,440 minutes from 242 videos of 94 children (ages 7–9 years) consuming four meals, spaced one week apart, with identical foods served in varying amounts. ByteTrack operates in two stages: (1) face detection via a hybrid Faster R-CNN and YOLOv7 pipeline, and (2) bite classification using an EfficientNet convolutional neural network combined with a long short-term memory (LSTM) recurrent network. The model was designed to handle blur, low light, camera shake, and occlusions (hands or utensils blocking the mouth). Performance was compared with manual observational coding.

**Results:**

On a test set of 51 videos, ByteTrack achieved an average precision of 79.4%, recall of 67.9%, and F1 score of 70.6%. Agreement with the gold-standard coding, assessed by intraclass correlation coefficient, averaged 0.66 (range 0.16–0.99), with lower reliability in videos with extensive movement or occlusions.

**Discussion:**

This pilot study demonstrates the feasibility of a scalable, automated tool for bite detection in children’s meals. While results were promising, performance decreased when faces were partially blocked or motion was high. Future work will focus on improving robustness across diverse populations and recording conditions.

**Clinical trial registration:**

https://clinicaltrials.gov/study/NCT03341247, identifier NCT03341247.

## 1 Introduction

Behaviors exhibited during a bout of eating (e.g., bites, chews, eating rate, bite-size, etc.) are collectively known as “meal microstructure.” Meal microstructure can be assessed to understand individual differences in eating patterns (1), the effects of food properties (1–3), and mechanisms of disordered eating and obesity (4–8). In pediatric populations, these insights are especially valuable for understanding obesity risk, as behaviors like larger bites and faster eating have been linked to greater food consumption and obesity (5, 8). Characterizing meal microstructure can provide insights into pediatric obesity risk, which could potentially lead to novel interventions to reduce this global pandemic (9). Observational studies have informed interventions targeting eating speed in children, with promising results. For example, an intervention (10) aimed at slowing child eating rates through educational materials and timers resulted in slower parent-reported eating rates and lower BMI gain over 8 weeks compared to the control group, suggesting interventions on meal microstructure hold promise for weight gain prevention in youth (11). Although these results are promising, research on meal microstructure may be held back by the expense and difficulty of reliably coding eating episodes. Developing methods like ByteTrack could help streamline measurement and improve the scalability and sustainability of this approach.

Several approaches have been developed to measure meal microstructure in humans. Currently, the gold standard for bite and microstructure analysis is manual observational coding (12), where researchers manually review videos and annotate bite timestamps through observation. Although observational coding is highly accurate and reliable (13), it is time-consuming, labor-intensive, and costly, making it less scalable and efficient compared to automatic bite detection systems. To address those limitations, wearable devices that use various sensor modalities such as acoustic sensors and accelerometers have been designed to record meal microstructure in adults (14). However, wearable sensor-based bite detection relies on predefined motion thresholds, which can lead to false positives (misidentifying hand movements like drinking or gesturing as bites), struggle with utensil variability (difficulty adapting to different eating methods such as chopsticks, spoons, or eating by hand), and face challenges in different contextual settings (15, 16).

To address challenges with wearable devices, several groups have developed automated approaches for bite counting from video, which are more adaptable. One method uses facial landmarks to define bites based on criteria like hand proximity or mouth opening (17–19). While effective in controlled environments, this approach is prone to false positives from non-eating behaviors such as gestures, talking, or facial expressions. Optical flow approaches (20, 21), which track motion between consecutive frames, also face significant limitations in reliably distinguishing between eating actions (i.e., bites) and other dynamic movements such as fidgeting, gesturing, or speaking. These challenges are especially pronounced in children (22, 23), who often engage in frequent hand-to-face movements or fidgeting, but similar issues can also occur in adults during social interactions.

Deep learning-based approaches (e.g., Convolutional Neural Networks or CNNs) have demonstrated stronger performance in bite detection (18, 20, 24) compared to facial landmark and optical flow-based models. However, these methods have primarily been tested under ideal conditions with high-quality video recordings of eating events, often involving adult participants. Real-world applications present a broader range of challenges, including varied lighting and higher variability in movement patterns, which are common across age groups. Development of deep learning approaches to automate bite detection would advance the field by making models more resistant to non-eating movements.

The purpose of this paper is to present ByteTrack, a deep learning model designed to detect bites and calculate eating speed in pediatric populations. To ensure its robustness in addressing challenges specific to pediatric samples, ByteTrack was directly trained on video recordings of children eating meals. It integrates advanced machine learning techniques, including Convolutional Neural Networks (CNNs) and Long Short-Term Memory-Recurrent Neural Networks (LSTM-RNNs). The main objectives of this paper are: (1) to develop a deep-learning-based bite detection system to automatically identify bites in video data recorded from children’s laboratory meals; (2) to evaluate ByteTrack’s accuracy and reliability by testing it on a designated video dataset (test set); and (3) to compare ByteTrack’s performance with manual (gold standard) annotations to assess its practical utility for capturing key measures of children’s eating behavior such as bite count, bite rate, and meal duration, along with correlations between measured intake with predicted bite counts.

## 2 Methods

### 2.1 Data collection

#### 2.1.1 Study design and participants

**Figure 1:**
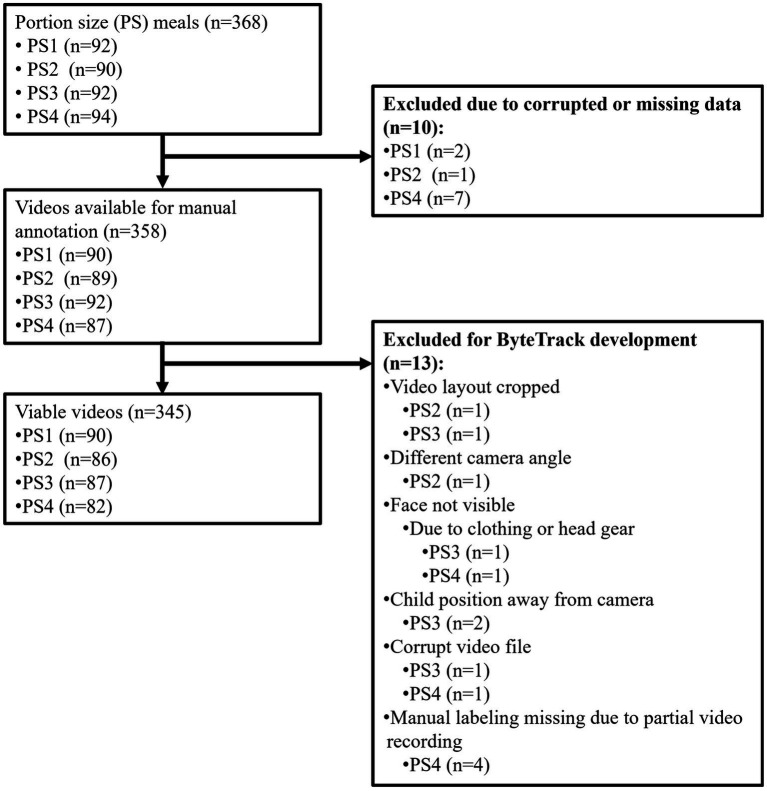
Flowchart of video dataset consideration for ByteTrack model development.

ByteTrack was trained on 242 videos (4,770 min) of laboratory meals in 94 children aged 7–9 years. Children consumed 4 laboratory meals with approximately 1-week between each meal. Video data came from the Food and Brain study (ClinicalTrials.gov, NCT03341247), a prospective investigation examining neural and cognitive risk factors for the development of obesity in middle childhood. The study employed a longitudinal family risk design, with children attending six baseline visits and one follow-up visit conducted 1 year after the first baseline visit. Data related to neurocognitive and longitudinal outcomes are reported elsewhere (25–31). Methods used to collect anthropometric and demographic data can be found in earlier studies (26, 27, 32). Families were recruited for the study based on maternal weight status: low (maternal BMI < 25) versus high (maternal BMI ≥ 30) risk for obesity. All children were below the 90th BMI-for-age percentile at baseline. The study received approval from the Pennsylvania State University Institutional Review Board. Parental consent and child assent were obtained at the first visit, and families received modest monetary compensation for completing each study visit. The final sample included 94 subjects who had a total of 345 viable meal videos (Figure 1 for reference to viability of data). Participant inclusion and exclusion with relevant CONSORT diagram are detailed in earlier publications (25). Demographic data for the sample can be found in Table 1. Supplementary Table 1 provides detailed information on subject-by-subject meal videos considered for training and testing, including subject IDs and meal sessions, along with details on exclusions and inclusions.

**Table 1:** Demographics of the sample.

Characteristic	Total Included (*n* = 94 children)		
Categorical variables			n (%)
	Sex		
		Male	49 (52.1)
		Female	45 (47.9)
	Race		
		White	91 (96.8)
		Non-white^#^	3 (3.2)
	Parental education^$^		
		<Bachelor’s degree (<16 years)	18(19.1)
		Bachelor’s degree (16 years)	42 (44.7)
		>Bachelor’s degree (>16 years)	34 (36.2)
	Parental income^†^		
		<$51,000	12 (12.8)
		$51,000–$100,000	45 (47.9)
		>$100,000	34 (36.2)

Continuous variables		Mean (SD), [min, max]	
	Age (years) at baseline	7.9 (0.6), [7.0, 9.0]	
	BMI percentile at baseline	47.9 (24.3), [3.9, 89.5]	

^#^Asian: *n* = 3; American Indian/Alaskan Native, Black/African American, Hawaiian/Pacific Islander: all *n* = 0; All participants reported not being Hispanic/Latino. ^†^Parent income missing values (*n* = 3). ^$^Parental Education: < Bachelor’s Degree (<16 years): High School/GED, Associate’s Degree, Technical or vocational school; > Bachelor’s Degree (>16 years): Master’s Degree, Ph.D.

#### 2.1.2 Laboratory meals

During visits 2–5, children were served meals of identical foods that varied by amount served (i.e., portion size), according to the aims of the parent study. The protocols for these meal sessions along with foods served have been previously described (25). In brief, meals consisted of foods that are common to United States children (i.e., macaroni and cheese, chicken nuggets, grapes, and broccoli). The reference portion (smallest) consisted of the usual serving sizes for this age group, and the subsequent three portions increased the amount served for each item by ~33%. The order in which children received the meals was randomly assigned and counter- balanced. Meal sessions were separated by at least a week.

Children had up to 30 min to eat *ad libitum* until comfortably full, while being read a non-food related story. Initially, these stories were read by a research assistant (*n* = 62). However, due to COVID-19 safety protocol changes, the remaining 32 children were either read a book by a parent (*n* = 16) or listened to a computerized audiobook (*n* = 16; Audible by Amazon, Newark, NJ). Intake was calculated by subtracting post-weight from pre-weight for each food. Intake was converted to kilocalories using Nutrition Facts panel Information or an online database.^1^

#### 2.1.3 Video recording

**Figure 2:**
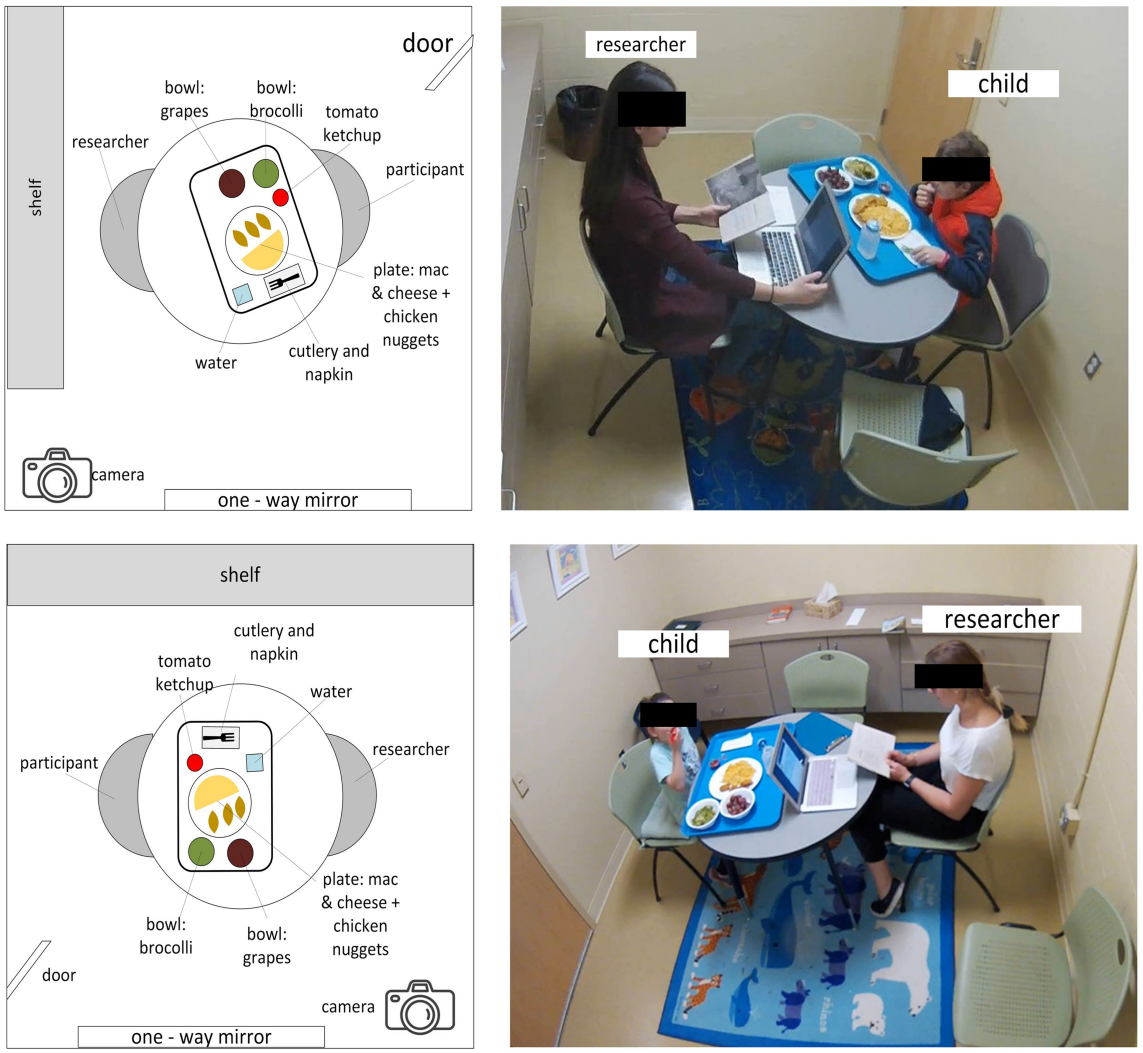
Observation room layouts for meal videos. Children’s meal intake was recorded in one of two observation rooms (A or B). Cameras were wall-mounted to record children’s meal intake.

Each eating event (meal session) was video recorded at 30 frames per second using an Axis M3004-V network camera. The camera was positioned outside the line of sight of children during the meal session. While parents were informed about the recordings, children were not. If a child noticed the camera or asked about its purpose, the research assistant explained that the camera was for safety purposes, aiming to reduce any observer effect. Figure 2 provides a schematic of the eating environment for the two dining rooms used over the visits, along with examples of a meal session.

### 2.2 Model building

**Figure 3:**
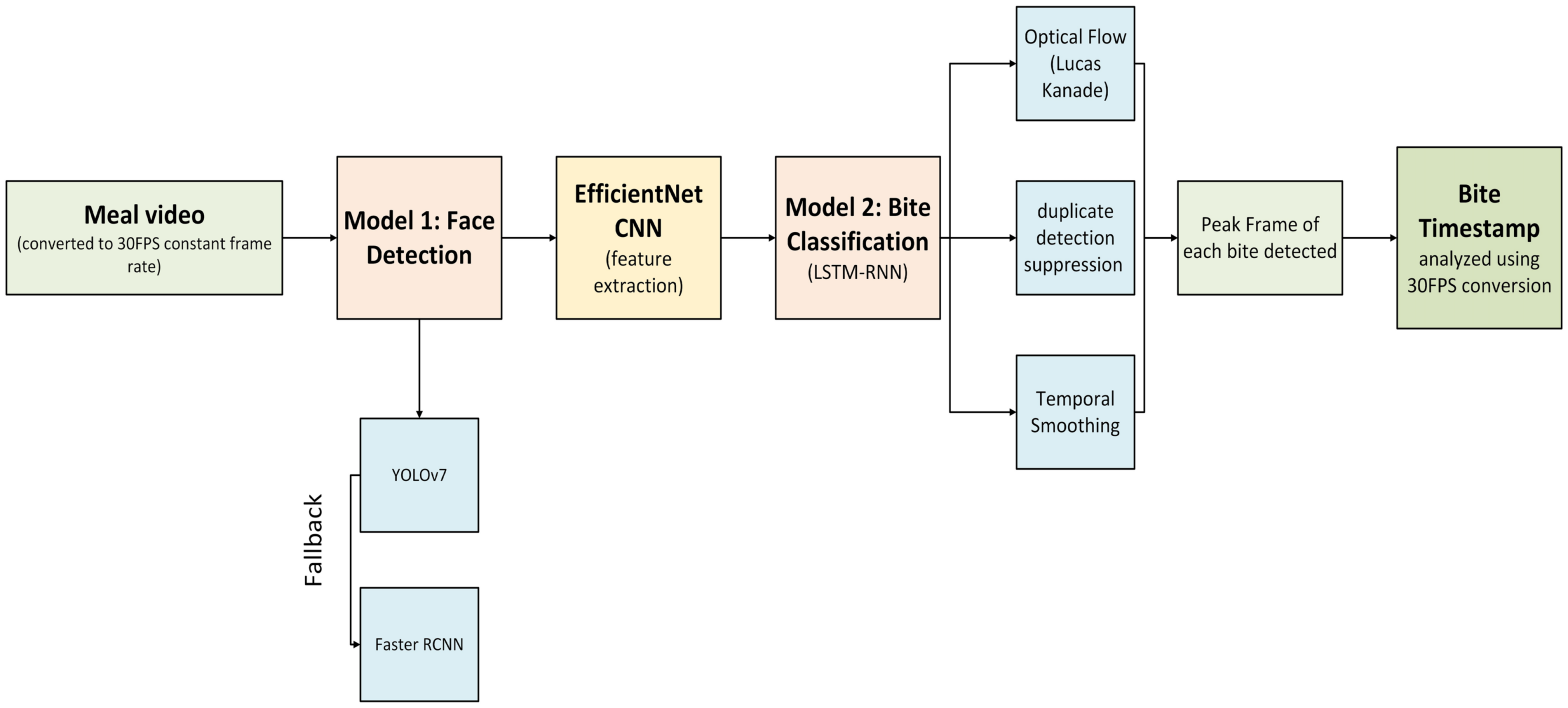
Overview of the bite detection pipeline. Meal videos were first converted to a constant frame rate of 30 frames per second (fps) before face detection (Model 1) using YOLOv7, with Faster R-CNN as a fallback. Detected faces were processed through EfficientNet CNN for feature extraction, followed by bite classification using an LSTM-RNN (Model 2). Post-processing techniques, including optical flow validation (Lucas-Kanade method), duplicate detection suppression, and temporal smoothing, were applied to refine predictions. The final output included the peak frame of each detected bite, with timestamps analyzed based on the 30fps conversion.

The ByteTrack pipeline for detecting bites consists of two major parts. The first part focuses on detecting and tracking faces in the video, to ensure that the system concentrates on the target child and ignored irrelevant objects or other individuals. The purpose of this is to reduce noise and prepare clean data for the second part of the pipeline. In the second part, the identified faces from the first step are analyzed to classify their movements and determine whether a child was taking a bite or performing other actions, such as talking or any irrelevant gestures. To enhance accuracy, a filtering process is applied to refine the results. Together, these steps form a 2-model pipeline to identify bites in videos. Diagrammatic representation of overall bite detection is in Figure 3. A more detailed flowchart for the system development is in Supplementary Figure 1. All model development, deployment, and statistical analyses were conducted using Python version 3.11.7 (33). Inter-rater reliability (ICC) was computed using Pingouin (v0.5.4) (34), while other statistical analyses, including regression and correlation, were performed using Statsmodels (v0.14.0) (35). F1 score calculations and classification metrics were computed using scikit-learn (v1.2.2) (36). Model development and computer vision tasks used PyTorch (v2.2.2) (37) and OpenCV (v4.5.3) (38). All plots and visualizations were generated using Matplotlib (v3.8.4) (39).

#### 2.2.1 Model 1: face detection

The first part of the model pipeline focuses on detecting and tracking faces in video footage, an essential step for identifying who is present and ensuring the system only analyzes relevant areas. To achieve this, we gathered a dataset of frames extracted from a random subset of videos to train and test the system. Two different approaches are used to detect faces: one that prioritizes speed for quick processing (You Only Look Once or YOLOv7) and another that is designed to handle more challenging situations (Faster Regional Convolutional Neural Network or Faster R-CNN), such as when faces are partially blocked or hard to see. The system is designed with the goal of achieving both efficiency and accuracy in face detection before progressing to Model 2 in the pipeline. Refer back to Figure 3 for an overview of ByteTrack model development.

##### 2.2.1.1 Step 1: model 1 dataset preparation

To ensure consistent frame rates across all videos for ground truth matching, meal videos (*n* = 345) are converted from a variable frame rate to a constant frame rate with .mp4 format and 30 frames per second (fps). This conversion is done using FFmpeg software (40) (version 4.3.2) with CUDA acceleration (h264_nvenc codec). Videos are then converted into frames at 6 frames per second to balance temporal resolution and computational efficiency.

A random sample of 52 videos (50 frames per video, 2,600 frames total) that include a diverse range of skin tones and seating positions is randomly selected for the face detection dataset. A 70–15-15% subject split is done (41, 42), yielding training (*n* = 1,800 frames, 36 subjects), validation (*n* = 400 frames, 8 subjects), and test (*n* = 400 frames, 8 subjects) sets. These sampled image frames are considered the Model 1 Dataset. To ensure an independent validation sample, no subjects appeared in more than one data set.

To enhance face detection model robustness to real-world variations in conditions (e.g., lighting changes, motion, camera angles, etc.,) the following augmentations are applied to the training dataset (43): Y-reflection (mirror), 2-D Gaussian smoothing (blurring), brightness adjustment, orientation change to portrait mode, and rotation (clockwise 10 degrees and anticlockwise 10 degrees). As a result of these adjustments, the total training set increased to include 10,800 images (i.e., frames of videos).

##### 2.2.1.2 Step 2: ground truth for model 1—manual face manual labeling

**Figure 4:**
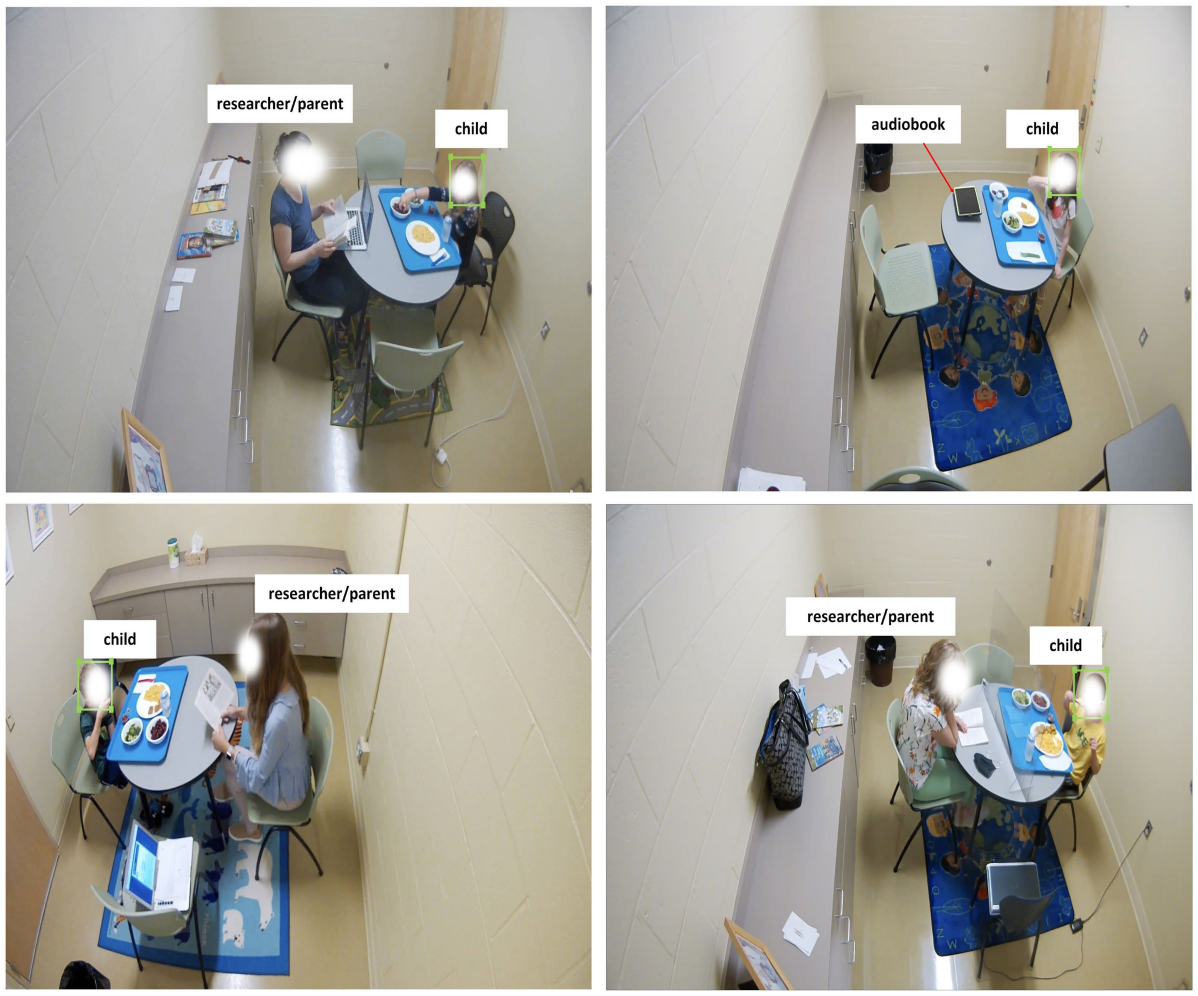
Examples of bounding boxes using LabelIMg used for labeling child faces for face detection.

To create a labeled dataset for training, within each image of the Model 1 Dataset, a single researcher (YRB) used bounding boxes to identify the children’s faces using the ImageLabeler API [LabelImg (44)]. Examples of the labeled images with bounding boxes are shown in Figure 4. The annotated label files are also augmented or transformed along with corresponding images in the training set. To maintain the aspect ratio (original size: 1,920 × 1,080) and reduce computation time, all original and transformed images are resized to 510 × 300 with padding (45) (when needed in augmentations). The Model 1 Dataset is utilized for both Faster R-CNN and YOLOv7 face detection models.

##### 2.2.1.3 Step 3A: automatic face detection with YOLO V7

YOLOv7 (46, 47) is used to develop a lightweight face detector to reduce computational costs. YOLOv7 is a one-stage object detection model designed for faster inference speeds compared to two-stage models like Faster R-CNN. In YOLOv7, the entire image is processed in a single pass through the network, allowing for faster and real-time object detection with lower computational overhead (47, 48). This makes YOLOv7 particularly suited for tasks that require fast detection in video-based settings.

YOLOv7 provides a general-purpose deep learning network for object identification, but the parameters must be fine-tuned to specifically identify children’s faces. To achieve this, the model was trained with Stochastic Gradient Descent (SGD) (49), a commonly used optimizer that updates model parameters incrementally on a subset of the training data, using an initial learning rate of 0.001 and exponential decay. The learning rate determines the size of the steps taken by the optimization algorithm (SGD) to adjust the model’s parameters during training, where a smaller learning rate makes smaller, more precise steps and a larger learning rate makes bigger, faster adjustments but risks overshooting the optimal solution. To balance early, faster progress with fine-tuning later in training, the learning rate was reduced gradually using an exponential decay (i.e., decreased by a fixed proportion over time), allowing the model to make smaller and more refined updates as training progressed. A batch size of four was used, meaning the model processed four samples at a time before updating its internal parameters. The training was conducted over 100 epochs, or complete passes through the entire dataset. To prevent overfitting, early stopping was applied to halt training if the validation set loss (a measure of model performance on unseen data during training) does not improve after 10 consecutive epochs (i.e., patience = 10). No additional hyperparameter tuning or cross-validation was performed beyond the standard training configuration provided by YOLOv7. Training was run on a Dell XPS 15 laptop with a 4GB GPU, 8 CPU cores, and 16GB of RAM. Training time was 6 h for YOLOv7 model.

##### 2.2.1.4 Step 3B. Automatic face detection with faster regional CNN

We used transfer learning on a Faster R-CNN model (50) with a ResNet-50 backbone (51) and Feature Pyramid Network (FPN) (52) for child face detection. Faster R-CNN is a two-stage object detection model that first generates regional proposals and then classifies and refines these regions, making it well-suited for tasks requiring accurate localization, such as face detection. The ResNet-50 backbone is a deep convolutional network with 50 layers, and its integration with FPN enhances the model’s ability to detect objects at multiple scales, which improves accuracy in detecting faces of varying sizes and positions.

To assess the model’s generalizability, we initially conducted 3-fold cross-validation (53), where the training set was split into three subsets or folds. Each fold used 7,200 training images and 3,600 validation images. Following cross-validation, we conducted a grid search (54) to fine-tune the model’s hyperparameters using the full training set, guided by feedback from a separate validation set. The model was trained using a batch size of 8 images with a loss accumulation over eight batches, which increased the batch size to 64 images. We used an initial learning rate of 0.01 with an SGD optimizer with a weight decay of 0.0005, a step size of 2 where the scheduler updated the learning rate every two epochs instead of default. We also used a momentum of 0.9 for the SGD optimizer to smooth parameter updates by incorporating a fraction of the previous update into the current one, where a high momentum (momentum = 1) gains all information from the previous step. Early stopping with a patience of three epochs was used based on feedback from the validation set for minimizing validation loss. Training was carried out on a high-performance cluster with 800GB RAM and 32 CPU cores. The training time with selected hyperparameters was 18 h and 22 min.

##### 2.2.1.5 Step 4: face detection using a combination of YOLOv7 and faster RCNN

We implemented a face detection and tracking system combining YOLOv7 and Faster R-CNN [similar to method used in (55)]. Videos were processed at 30 fps, with YOLOv7 handling initial detection. If YOLOv7’s confidence score exceeded 0.8, its detected bounding box was used to track the child’s face using a Kernelized Correlation Filter (KCF) tracker (56), a high-speed tracker which updated every 20 frames to maintain accurate localization and prevent drift.

YOLOv7 served as the primary detector, provided it successfully detected a face with a confidence score of 0.8 or higher. If YOLOv7 failed to detect a face or produced a confidence score below this threshold, Faster R-CNN was used as a fallback. By default, the detections were weighted, with YOLOv7 assigned 80% and Faster R-CNN 20%. However, if YOLOv7’s bounding box was significantly smaller—less than 30% of the area of the Faster R-CNN detection—the weights were adjusted to 60% for YOLOv7 and 40% for Faster R-CNN. This approach leveraged YOLOv7’s speed while incorporating the robustness of Faster R-CNN for more reliable detection. Detected face images were resized to 224×224 pixels to prepare images for Model 2, which aimed to detect and classify bites.

#### 2.2.2 Model 2: bite classification

For Model 2, we aimed to accurately classify bite events by leveraging deep learning on high-level facial features. This involved training a sequential model (LSTM) using manually annotated bite data while addressing class imbalance and optimizing classification performance. Post-processing techniques were applied to refine detections and minimize false positives, ensuring better accuracy for bite event identification from video data. Refer back to Figure 3 for an overview of ByteTrack model development.

##### 2.2.2.1 Step 1: ground truth for model 2—manual annotation of bites

Manual annotated timestamps were used as ground truth for model training of bite instances. Coding was conducted using Noldus Observer XT v16 (Noldus, 1991). Bites of food, sips of water, and active eating time were coded using an established protocol developed by Pearce and colleagues (12, 57, 58). All videos were coded by two independent research assistants. The inter-rater reliability for each behavior, calculated using intraclass correlation coefficients [ICC (1, 3) i.e., two-way mixed-effects model for a single measure (59)] was excellent for all eating events, ICCs >0.98 (25).

##### 2.2.2.2 Step 2: pre-processing for bite classification (model 2)

As described previously, detected face images were resized to 224×224 pixels to prepare images for the next steps, which included feature extraction through Efficient Net Convolutional Neural Network (EfficientNet CNN) and bite classification through Long Short-Term Memory Recurrent Neural Network (LSTM-RNN). Bites were tagged with a timestamp in seconds to map each detection to the corresponding video frame.

**Figure 5:**
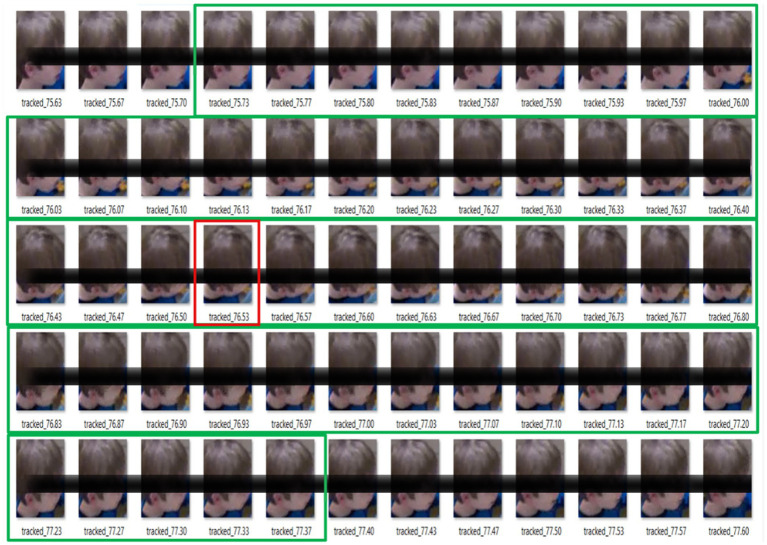
Example of bite labeling; bite center marked in red and whole bite sequence marked in green.

For bite classification, all videos (345 videos) were split into training, validation, and test sets (70–15-15%) (41, 42) while maintaining split consistency with the Model 1 Dataset (from face detection split). An average bite sequence was assessed to be 50 frames through visual inspection (i.e., ~1.7 s). Bite sequences were selected as 50 frames with the manually annotated timestamp placed at the center (i.e., 25th frame). Non-bite sequences were selected with a 10-frame buffer between bite and non-bite sequences. An example of bite sequence labeling is shown in Figure 5.

Bite sequences with more than 45 valid frames (≤10% missing data) were retained and padded to retain constant sequence length of 50 frames. Padding involves adding placeholder frames, here all-black frames, to ensure all sequences have a consistent length to facilitate uniform processing and analysis. Masking was applied to ensure that the LSTM ignores padded frames, preventing it from learning patterns from missing or non-informative data (60). Any sequences with fewer than 45 frames (>10% missing frames) were discarded. The resulting training set contained 13,527 bite sequences (minority class) and 77,653 non-bite sequences (majority class).

###### 2.2.2.2.1 Addressing class imbalance and loss function

Class imbalance is a common challenge in visual classification tasks, including food-related applications (61). To address the significant class imbalance between bite (minority class) and non-bite (majority class) events, we implemented a hybrid sampling approach and a custom loss function (error minimization function; detailed in Supplemental material). This method combines random undersampling of the majority class (62) and Synthetic Minority Over-sampling Technique (SMOTE)-based oversampling of the minority class (63) to preserve as much information as possible. This combination approach simultaneously reduces the risk of information loss from extreme undersampling of the majority class and prevents redundancy from excessive oversampling of the minority class. The majority class (non-bites) was undersampled to 3x the size of the minority class (bites), where the undersampling ratio was chosen through grid search. After undersampling, we had 13,537 bites and 40,611 non-bite sequences. Next, the bite class was oversampled to match the non-bite class sample size using SMOTE (63). The final Model 2 Dataset had 40,611 bite sequences and 40,611 non-bite sequences.

##### 2.2.2.3 Step 3: bite vs. non-bite classification

We implemented a bite classification model using transfer learning with EfficientNet-CNN (64), a lightweight convolutional neural network designed for image recognition. Bite and non-bite frame sequences were passed through the encoding layers of EfficientNet-CNN (but not the classification layer) to transform the images into a set of high-level nonlinear features. These features, along with their corresponding labels and masks, were then fed into an LSTM-RNN (65), a commonly used time sequence model that can intake a sequence of images for action detection (i.e., bite classification).

###### 2.2.2.3.1 Training parameters for LSTM bite classification

A bidirectional LSTM, a variation of LSTM that gathers information from both the beginning and end of a sequence, was used for bite detection. The grid search (54) identified the optimal hyperparameters as follows: a batch size of 128, a learning rate of 5 × 10^−5^, 40 training epochs, three hidden layers, and hidden size as 256. To reduce overfitting, we applied a dropout rate of 0.4 during training. This reduces dependence on any single feature and helps the model generalize better to unseen data. The model was trained using the Adam optimizer (66), selected for its adaptive learning rate capabilities and computational efficiency. Early stopping was employed based on validation F1 score improvement with patience = 5.

Dynamic thresholding was used during training to adjust the model’s confidence level for each bite prediction. The model tested different thresholds, adjusting the point at which a prediction is considered correct (i.e., when the model is confident enough to label an event as bite). After testing multiple thresholds, 0.65 was determined to be the optimal value, providing the best balance between minimizing false positives and false negatives in the bite detection task. This ensured the model could detect bite events accurately without over- or under-predicting. Training, validation, and testing were conducted on a high-performance cluster with 800GB RAM and 32 CPU cores. The training time with selected hyperparameters was 26 h and 35 min.

##### 2.2.2.4 Step 4: automatic bite detection from video

*Post hoc* processing was applied to enhance bite detection precision using three techniques: temporal smoothing (67), duplicate detection suppression (68), and optical flow validation (69). Temporal smoothing was achieved by applying a moving average over a 20-frame window to stabilize detection probabilities and reduce noise from transient movements. The purpose of this smoothing was to reduce noise and allow for detection of trends in the data. To prevent overcounting the same bite event, we enforced a 15-frame interval threshold, filtering out additional detections within this period. This threshold ensured that each detection was distinct, allowing for improved accuracy by spacing out events and reducing duplicates. Optical flow validation was employed to further reduce false positives. Using the Lucas-Kanade method (69), key points were tracked over a 2-s window post-bite to confirm chewing motion. A small motion threshold of 0.02 ensured that detected events exhibited the typical small, repetitive motion of chewing, filtering out unrelated movements. Bite detection was conducted on a Dell XPS 15 laptop with a 4GB GPU, 8 CPU cores, and 16GB of RAM.

### 2.3 Model performance

The ByteTrack pipeline was evaluated based on its accuracy and reliability in detecting bites from video data. Performance was assessed by testing the model on a designated video dataset (test set) and analyzing key metrics. ByteTrack’s bite predictions were compared with manual annotations (*n* = 51 videos) using both Pearson correlation and simple linear regression. Correlation was used to assess the strength of association, and regression was used to evaluate the linear fit and prediction error for bite count and meal duration. Agreement between methods was further evaluated using ICC and Bland Altman analysis.

Simple linear-regression coefficients (intake ~ bite count) were computed to relate predicted bite counts to measured intake (*n* = 50 videos; *n* = 1 excluded for missing intake).

The overall ByteTrack pipeline with face detection (Model 1) followed by bite classification (Model 2) were utilized for bite classification and identifying the timestamp at which the bite occurred in the video. For bite timestamping, we selected the frame with the peak detection probability over each event, which was then converted to seconds using a 30 FPS frame rate. To accommodate computational delay, a 10-s margin around each manual timestamp was applied, marking detections within this window as true positives (TP). This margin accounts for annotation variability, temporal smoothing effects that may shift predictions, and the focus on bite count over exact timing. Since bite detection prioritizes detecting the correct number of bites rather than precise frame-level accuracy, this margin ensures a fairer evaluation aligned with real-world use cases. Missed manual bites were labeled false negatives (FN), and extra detections by the model were false positives (FP). Similar to the individual model performance, the same metrics—precision, recall, and F1 score were calculated for each video in the test set. The overall precision, recall, and F1 scores were found by taking the arithmetic mean for all 51 videos. Detailed information on video-to-video on performance metrics is available in Supplementary Table 2.

#### 2.3.1 Model performance metrics

Model 1 and Model 2 were tested individually on their respective test sets to calculate common performance metrics, such as precision, recall, and F1 score.

(1) Precision, which indicates how many detected bites were true and helps assess false positives, was calculated as the proportion of detected bites that were actual events.

$$\text{Precision}(\%)=\frac{\text{True Positives}\;(\mathrm{TP})}{\text{True Positives}\;(\mathrm{TP})+\text{False Positives}\;(\mathrm{FP})\;}\times 100\%$$

(2) Recall assessed the ability to identify all actual bite events by calculating the proportion of true bites correctly detected:

$$\text{Recall}(\%)=\frac{\text{True Positives}\;(\mathrm{TP})}{\text{True Positives}\;(\mathrm{TP})+\text{False Negatives}(\mathrm{FN})\;}\times 100\%$$

(3) F1 score provides a balanced assessment of the model’s ability to accurately detect bites while minimizing false detections by calculating the harmonic mean of precision and recall:

$$F1\;\text{score}(\%)=2\times \frac{\text{Precision}\times \text{Recall}}{\text{Precision}+\text{Recall}}\times 100\%$$

#### 2.3.2 Inter-rater reliability

To assess the reliability of automated bite detection, we used the Intraclass Correlation Coefficient (ICC) (59), a two-way mixed-effects model for a single measure. This model, appropriate for a fixed set of raters (one of the human raters for ground truth and the automated detection model, ByteTrack), evaluated consistency in bite event identification. Calculating ICC provided a measure of agreement between ByteTrack and human annotations, focusing on consistent detection across repeated measures within each subject. We then averaged ICC values across subjects to assess the overall reliability of the model’s performance across the dataset.

#### 2.3.3 Assessment of model eating behavior detection

Multiple metrics were used to assess systematic errors and overall performance of the ByteTrack bite count and meal duration predictions against manual ground truth. Scatterplots were employed to visualize the relationship between modeled and manual metrics and identify trends in overestimation or underestimation and quantitative measures such as Root Mean Square Error (RMSE), percentage RMSE (%RMSE), and error percentage (Error %) capture the magnitude and nature of deviations. Additionally, to understand the relationship of predicted bite count with actual intake (*n* = 50, 1 participant excluded due to unavailability of objective intake measure), correlations between predicted bite count and measured energy intake (kcal) and gram intake at meals were calculated. The specific metrics were:

(1) Slope, which reflects proportional errors, with values >1 indicating overestimation and <1 indicating underestimation with a 45° line (y = x) representing perfect agreement.
(2) Intercept, which reflects any consistent bias or offset.
(3) R^2^, which provides an overall measure of how well the modeled values explain the variance in manual values.
(4) RMSE, which assesses raw error while maintaining units by calculating the average deviation between predicted and manual metrics (i.e., RMSE tells how far off the model is on average from the true values):

$$\text{RMSE}=\sqrt{\frac{1}{N}\sum_{i=1}^{N}{\left(y_{i}-{\hat{y}}_{i}\right)}^{2}}$$

where $y_{i}$ = manual value, ${\hat{y}}_{i}$ = modeled value, N = total number of observations.

(5) RMSE%, which allows for comparison between metrics by normalizing RMSE relative to the mean of the manual metrics:

$$\text{RMSE}\%=\frac{\text{RMSE}}{\bar{y}}\times 100\%$$

where $\bar{y}$ = mean of the manual values, calculated as $\bar{y}=\frac{1}{N}\sum_{i=1}^{N}y_{i.}$

(6) Error %, which assesses localized patterns of bias in the model’s predictions by calculating the deviation between predicted and actual bite counts across different videos.

$$\text{Error}\%=\frac{\left({\hat{y}}_{i}-y_{i}\right)}{y_{i}}\times 100\%$$

where $y_{i}$ = manual value, ${\hat{y}}_{i}$ = modeled value

We conducted a retrospective visual review of the videos to gain a general understanding of where the model performed well or poorly. This visual inspection aimed to estimate potential reasons for mismatches in bite count and meal duration between the model and manual annotations.

## 3 Results

### 3.1 Model performance

#### 3.1.1 Model 1—face detection

Both YOLOv7 and Faster RCNN were evaluated using an Intersection over Union (IoU) threshold of 0.5, a standard measure in object detection. An IoU threshold of 0.5 means that a prediction is considered correct if the predicted bounding box overlaps with at least 50% of the actual object’s (manually labeled) bounding box. This threshold is widely used because it provides a balanced approach to precision and recall, ensuring predictions are accurate without being overly strict. A threshold lower than 0.5 might allow too many false positives, while a higher threshold could miss valid detections that are not perfectly aligned.

YOLOv7 achieved a precision of 98.12%, recall of 94.35%, and an F1 score of 96.98%. These results demonstrate YOLOv7’s ability to detect faces quickly and accurately. YOLOv7 also shows slightly lower recall (i.e., misses some faces). Faster RCNN had a precision of 92.94%, recall of 98.75%, and an F1 score of 95.76%, with higher recall than YOLOv7. We therefore used the faster model YOLOv7 as the primary model with Faster RCNN as a fallback.

This combination of YOLOv7 as primary model with Faster RCNN as fallback, gave us a precision of 99.24%, recall of 98.25%, and F1 score of 98.74% at an IoU = 0.5.

#### 3.1.2 Model 2—bite classification

The LSTM-RNN model achieved a mean precision of 72.8%, mean recall of 80.9% and an mean F1 score of 76.2% for bite detection across the test dataset (*n* = 51 videos). This performance was evaluated on a test set comprising 3,776 bite sequences and 22,140 non-bite sequences in a sequence-to-sequence analysis at a confidence threshold of 0.65. This is a sequence-to-sequence analysis, i.e., measuring performance on chunks of image sequences (images from Model 1), which allows for a controlled evaluation of the model’s bite classification ability, independent of continuous video tracking errors, frame inconsistencies, and temporal noise. By focusing on pre-segmented sequences derived from object detection, this approach isolates the LSTM’s performance, ensuring that the assessment reflects its ability to recognize temporal patterns without the confounding effects of tracking stability.

#### 3.1.3 ByteTrack performance—bite detection

ByteTrack’s bite detection performance was evaluated on 51 videos from 42 children, achieving an average precision of 79.4%, recall of 67.9%, and an F1 score of 70.6% with a 10-s margin from ground truth. We see large variability between subjects, with precision ranging from 38.2 to 100%, recall from 17.6 to 93.6%, and F1 score from 26.3 to 91.2%. Post-hoc smoothing likely improved precision by filtering spurious detections but reduced recall by removing some true bites. The confusion matrix from the ByteTrack system on the test set (*n* = 51 videos) is shown in Table 2.

**Table 2:** Confusion matrix on test set for bite detected in test video data using ByteTrack (*n* = 51 videos).

		Predicted classes	
		Bite	Non-bite
Actual class	Bite	5,213 (TP)	1,842 (FN)
	Non-bite	1,653 (FP)	Unknown (TN)

TP, True positives; FP, False positives; TN, True negatives; FN, False negatives.

Although we did not log inference time per video, ByteTrack typically processed a 30-min video in ~25–30 min on a Dell XPS 15 laptop (4GB GPU), depending on activity level. In contrast, manual double-coded annotation took ~70–80 min per video.

### 3.2 ByteTrack performance relative to gold-standard manual annotation

#### 3.2.1 Inter-rater reliability

The reliability of bite events between manually coded data and ByteTrack or inter-rater reliability, measured using ICC, showed moderate reliability (59) with a mean value of 0.66 and a range of 0.24–0.99.

#### 3.2.2 Bite count

**Figure 6:**
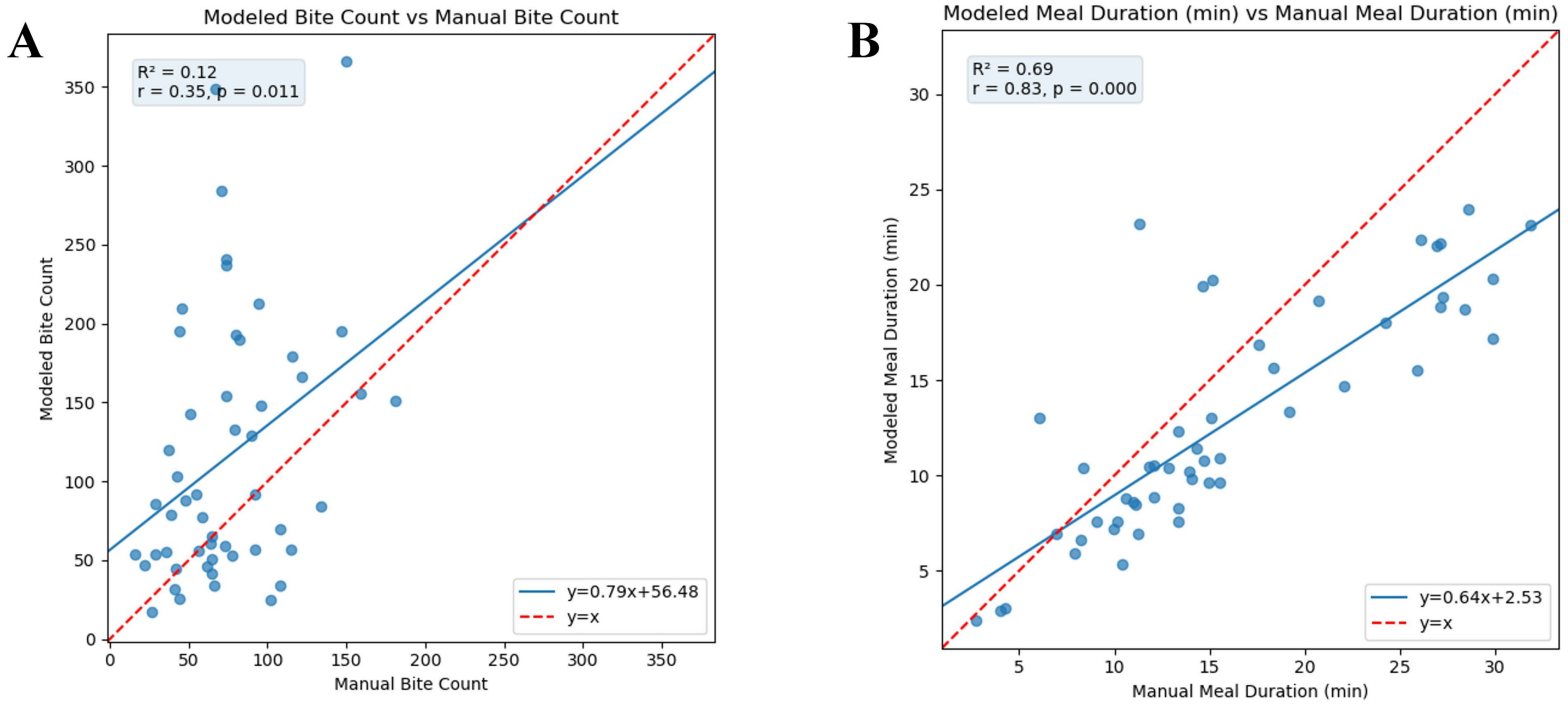
Scatter plots showing the relationship between manual (ground truth) and modeled (predicted by ByteTrack) eating behavior metrics (*n* = 51 videos), assessed using both Pearson correlation and simple linear regression. The red line represents ideal agreement (y = x), and the blue line shows the fitted regression line. Each point represents one test video. **(A)** Manual vs. modeled bite count. **(B)** Manual vs. modeled meal duration. RMSE, Root Mean Square Error, measures the average magnitude of prediction error; RMSE%, RMSE expressed as a percentage of the mean manual value; Error%, Average absolute percentage difference between manual and modeled metrics; R^2^, Coefficient of determination from the regression model; r, Pearson correlation coefficient.

The scatter plot comparing modeled and manual bite counts shows consistent overestimation by the model (Figure 6A). Linear regression fitted across all data points produced a slope of 0.79 and an intercept of 56.48, with an R^2^ of 0.12 and a Pearson correlation coefficient of *r* = 0.35, indicating a weak linear association between modeled and manual counts. The mean of the per-subject RMSE was 61.6 bites and mean per-subject RMSE% of 96.9%. The mean per-subject error% was 72.9%. The model captures the general bite count trend, with predicted counts correlated to ground truth. It overestimates on average and shows high variability across subjects. Children with higher true bite counts are generally ranked higher, despite errors in exact values. A Bland–Altman plot depicting the differences in bite counts can be found in Supplementary Figure 2. The Bland–Altman plot shows that model-predicted bite counts were on average 47.6 bites higher than manual counts, with 95% limits of agreement ranging from −197.8 to 112.6 bites (manual—model), indicating that differences between the two methods spanned from the model predicting more bites to the manual count exceeding the model. The differences appear to widen with increasing average bite counts.

#### 3.2.3 Meal duration

The relationship between manual and model-calculated meal duration is shown in Figure 6B indicating a moderate positive relationship between modeled and manual durations. A linear regression fitted across all data points produced a slope of 0.64 and an intercept of 2.53 with an R^2^ of 0.69, indicating moderate linear association between modeled and manual computed meal duration. The mean of the per-subject RMSE was 4.39 min, with a mean per-subject RMSE% of 28.3%. The mean percentage error, derived from the percent error per subject, was −16.0%, indicating a systematic underestimation.

#### 3.2.4 ByteTrack performance with real-world eating behavior outcomes

**Figure 7:**
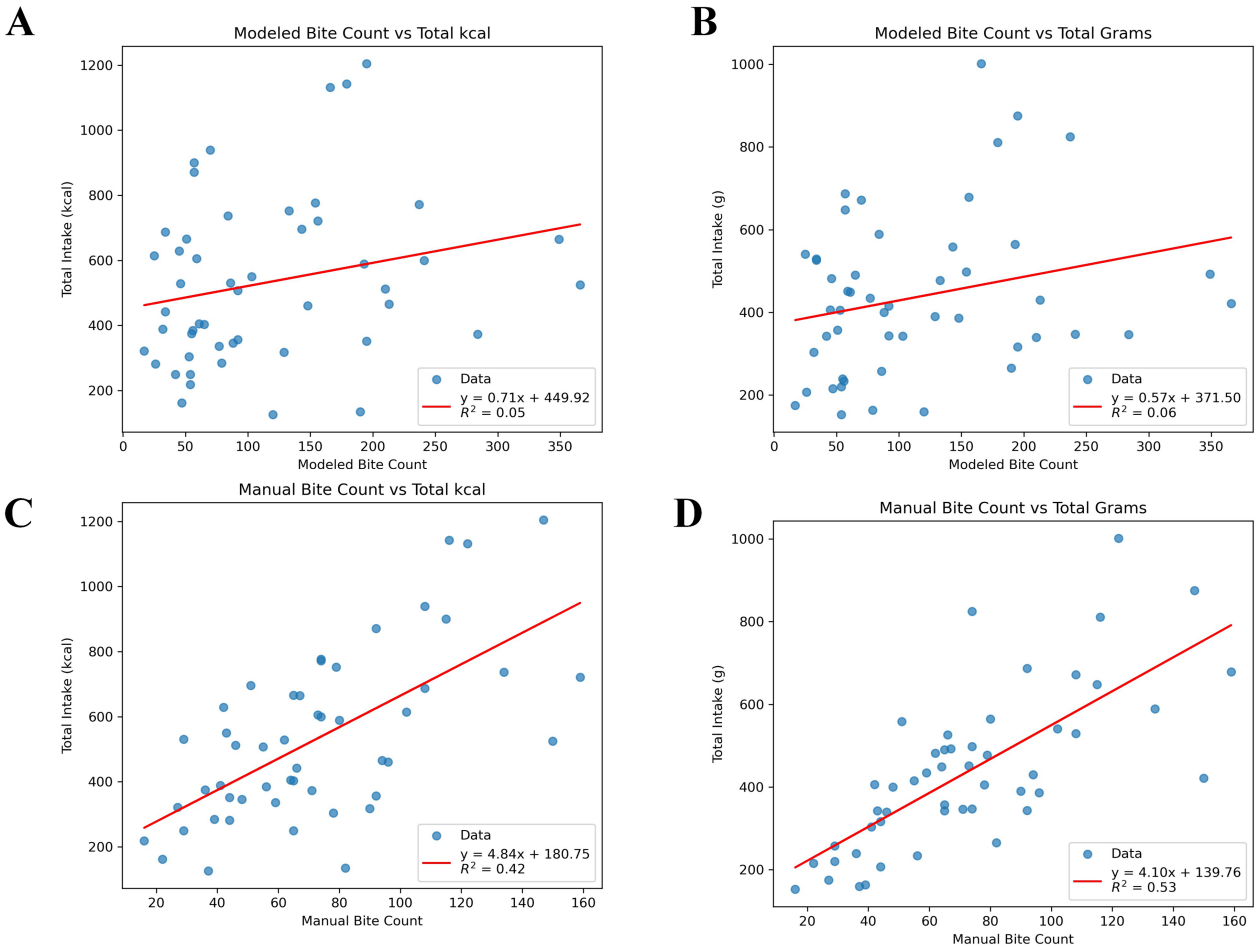
Scatter plots showing the correlation between modeled or predicted bite counts **(A,B)** and manual or ground truth **(C,D)** vs. actual meal intake (grams or kcal; *n* = 50 videos; *n* = 1 missing measured meal intake). **(A)** Scatter plots showing modeled bite counts vs. actual calculated energy (kcal) intake at meal. **(B)** Scatter plots showing modeled bite counts vs. actual calculated gram intake at meal. **(C)** Scatter plots showing manual bite counts vs. actual calculated energy (kcal) intake at meal. **(D)** Scatter plots showing manual bite counts vs. actual calculated gram intake at meal. R^2^, coefficient of determination from simple linear regression (intake ~ bite count).

Simple linear regression models were used to assess the ability of ByteTrack to model eating behavior that relates to real-world outcomes such as meal intake. The relationship between meal energy intake (kcal) and gram intake (g) and modeled bite counts is shown in Figures 7A,B respectively. The relationships between meal energy and gram intake with the manual annotations are in Figures 7C,D. The relationship between modeled bite count and meal intake shows weak but clear trends. The R^2^ values (regression coefficient) are low (*R*^2^ = 0.05 for kcal, *R*^2^ = 0.06 for grams), with high variability in how much modeled bite count predicts intake. Both figures show positive slopes (i.e., higher bite count associated with higher intake) between modeled bite counts and measured intake. Substantial inter-individual variability is seen in the plots. Positive slopes in both figures show higher bite counts are generally associated with greater intake. While the associations are weaker than those observed with manual bite counts (*R*^2^ = 0.42 for kcal, *R*^2^ = 0.53 for grams), the trends remain evident, suggesting that modeled bite count captures meaningful intake patterns despite variability across individuals.

## 4 Discussion

ByteTrack demonstrated moderate performance, with an average F1 score of 71% and an inter-rater reliability (ICC = 0.66) when compared to manually annotated ground truths. To our knowledge, this is the first automated system specifically developed to analyze eating behaviors in children, whose video data presents unique challenges due to frequent movements and occlusions. ByteTrack serves as a proof-of-concept for automated bite detection in children and suggests a promising future for this direction of research.

To support robust bite detection, the first stage (part 1) of the pipeline focused on accurate face localization despite child movement and occlusion. A two-stage detection strategy was used, in which a fast, high-precision YOLOv7 model served as the primary detector, while a higher-recall Faster R-CNN acted as a fallback in cases of missed detections. This design allowed the system to maintain high face detection performance (recall and precision >98%), balancing the need for speed with tolerance to the visual variability common in child mealtime videos.

Bite detection (part 2) showed greater variability but moderate performance across subjects (mean F1 = 71.3%; ICC = 0.66). However, total overall bite counts were generally inflated, with over-firing concentrated in the early portion of meals and under-firing during later or longer sessions. Retrospective visual inspection of videos suggests several possible contributors. Rapid, closely spaced bites, often involving brief spoon nibbling, may blur event boundaries and lead to extra detections. As meals progress, children tend to shift focus or play with food, producing more body movement and occlusions that can suppress detections. Together, these factors appear to let the model identify the general timing of bites yet trigger too frequently around true events at the start and too sparingly as eating slows, leading to shorter estimated meal durations.

While the video data used for ByteTrack was collected in controlled laboratory settings, the conditions of recording simulated a more natural mealtime environment in that additional people were present to engage with children (~80% videos in training data and ~82% videos in test set with additional person). This approach contrasts with previous systems developed for adults in tightly controlled settings (18, 20) by accommodating the unique behavioral patterns and interactions typical in a child’s meal. However, the majority of a child’s food intake at this age takes place at home and school (70–72), therefore future studies are needed to improve the flexibility of ByteTrack to evaluate eating behaviors in these diverse settings.

Traditional assessment methods for eating rate rely on self-reporting, which is often inaccurate due to memory lapses and social desirability bias (73, 74). More objective measurements come from wearable devices and video-based monitoring. Wearable devices, such as bite counter watches (75) and sensor-based eyeglasses (76), can track bites. But there are limitations with these devices as they require researchers or users to start and stop data collection which can be intrusive to the natural eating process. Video-based monitoring methods like ByteTrack, while also requiring similar manual start/stop, offer a less intrusive approach for measuring meal eating behaviors that aligns with current gold standards of manual observational coding. Accurate, automated, real-world video-based approaches may enable the use of smartphone cameras for passive dietary monitoring in naturalistic settings, such as at home, creating new opportunities for scalable dietary data collection and intervention. Applying home recording methods with ByteTrack for automated bite detection provides a practical solution for capturing meal and snack intake, enabling the estimation of food intake and eating rates in natural settings. However, as these technologies advance, ensuring data privacy and encryption will be critical for secure handling of sensitive information (77).

The goal for future iterations of ByteTrack will be to replace or supplement manual observational coding, as it is a highly time and resource-intensive process. In the current study, double-coded manual annotation took approximately 80 min per 30-min video (>40 h total for the dataset), posing challenges for scaling to larger datasets. In contrast, ByteTrack completed the same task in typically 25–30 min per 30-min video, with minimal human input beyond initiating the script. However, this version of ByteTrack is not yet optimized for real-time bite detection. Human annotators may also introduce variability due to differences in interpretation, fatigue, or experience, which is why double coding is used to ensure reliability by resolving discrepancies between two independent annotations. In contrast, automated coding can apply consistent criteria across all videos, eliminating the need for double coding and improving research efficiency. Once refined, automated approaches like ByteTrack could enhance the ability to study human eating behavior outside the laboratory.

While the ByteTrack model had moderate F1 scores, which demonstrated good alignment with manual annotation, performance variability of the model highlights areas for improvement in future iterations. The ByteTrack system was less accurate when children are rapidly and had pronounced head and hand movements, potentially leading to a higher number of false positives (i.e., mistaking these movements for bites). False negatives occurred when bite motions were occluded, such as when a child’s hand, utensils, or other objects blocked the view of their mouth, which led to missed bite events and lower recall in detection accuracy. Additionally, it appears that the model overestimates the bite count during the initial rapid eating phase, when the child is more focused on eating. As the meal progresses and becomes longer, the child may slow down, move around, or lose attention to the food, leading to increased occlusions and missed bite events. This shift could result in the overestimation of bite counts early in the meal and the underestimation of meal duration later on, as fewer bites are detected when eating slows and occlusions become more frequent. This pattern of overestimating bite counts at the start of longer meals and underestimating meal duration in the later stages seems to contribute to overall inaccuracies in both bite count and meal duration estimation. Another limitation of ByteTrack is that there was no explicit modeling to differentiate bites from sips, which could have led to a misclassification of sips as bites. These challenges underscore the need to further refine the ByteTrack model to enhance its robustness in naturalistic eating scenarios.

Despite its limitations, there are several strengths to the current iteration of ByteTrack. As a non-intrusive, video-based system, it provides an alternative to wearable sensors and sets the stage for large-scale, automated detection by reducing reliance on manual annotation. The deep learning architecture used to construct ByteTrack combined state of the art methods (e.g., YOLOv7, Faster R-CNN, and LSTM) to achieve accurate face detection while accounting for the unique movement patterns of children. Furthermore, extending ByteTrack’s application beyond the lab to home and school environments, a direction in our ongoing studies, will further validate ByteTrack’s performance and enhance its real-world applicability.

Future iterations of ByteTrack will enhance robustness by incorporating diverse training data, including varied lighting conditions, movement patterns, and occlusions (78). Action detection in real-world video remains challenging due to the variability in human movement and environmental conditions (e.g., lighting, cluttered backgrounds). Data augmentation techniques, such as occlusion augmentation (e.g., adding synthetic hands, utensils, or objects partially covering the mouth), motion blur to reflect natural head movements, and temporal adjustments like varying frame rates or inserting brief distractions, can help simulate real-world eating scenarios (45). Additionally, integrating inter-subject variability by using subject identity as a model feature can improve the system’s ability to distinguish bites from non-bites (79). Explicitly modeling bite and sip classification separately may improve accuracy and reduce bite overestimation in the current model. Incorporating more data from real-world smartphone videos may further enhance performance and practical utility. Moreover, ByteTrack’s bite-count output could also be paired with complementary tools for portion-size estimation and food identification (80, 81) to yield more precise, holistic measures of meal microstructure and dietary intake in future work.

ByteTrack is a proof-of-concept, automatic bite detection framework for easing the time and resources required for manual video annotation. This represents a first step toward scalable, automated bite detection for the measurement of meal-related eating behaviors in children. With additional testing and model improvements, ByteTrack may expand the ability to capture real-time changes in human eating behaviors measured outside the laboratory.

## Data Availability

The datasets presented in this study can be found in online repositories. The names of the repository/repositories and accession number(s) can be found at: https://github.com/YashuBhat96/ByteTrack and https://osf.io/g6muv/.

## Footnotes

^1^ https://fdc.nal.usda.gov/

## Glossary

AI: Artificial Intelligence
API: Application Programming Interface
CNN: Convolutional Neural Network
CPU: Central Processing Unit
COVID-19: Coronavirus Disease 2019
F1: F1 Score (harmonic mean of precision and recall)
FN: False Negative
FP: False Positive
FPS: Frames Per Second
FPN: Feature Pyramid Network
GPU: Graphics Processing Unit
ICC: Intraclass Correlation Coefficient IoU Intersection of Union
KCF: Kernelized Correlation Filter
LSTM: Long Short-Term Memory
MP4: MPEG-4 Video Format
R-CNN: Regional-Convolutional Neural Network
RAM: Random Access Memory
ResNet: Residual Network
RMSE: Root Mean Square Error
RMSE%: Percentage Root Mean Square Error
RNN: Recurrent Neural Network
SGD: Stochastic Gradient Descent
SMOTE: Synthetic Minority Over-sampling Technique
TN: True Negative
TP: True Positive
YOLOv7: You Only Look Once, version 7
