# Supplementary material for: ByteTrack: a deep learning approach for bite count and bite rate detection using meal videos in children
Source: Front Nutr. 2025 Oct 3;12:1610363. doi: 10.3389/fnut.2025.1610363 (PMC12532775; doi:10.3389/fnut.2025.1610363)
Supplement: Supplementary file 1 [file Table_1.DOCX]

**Supplementary Text**


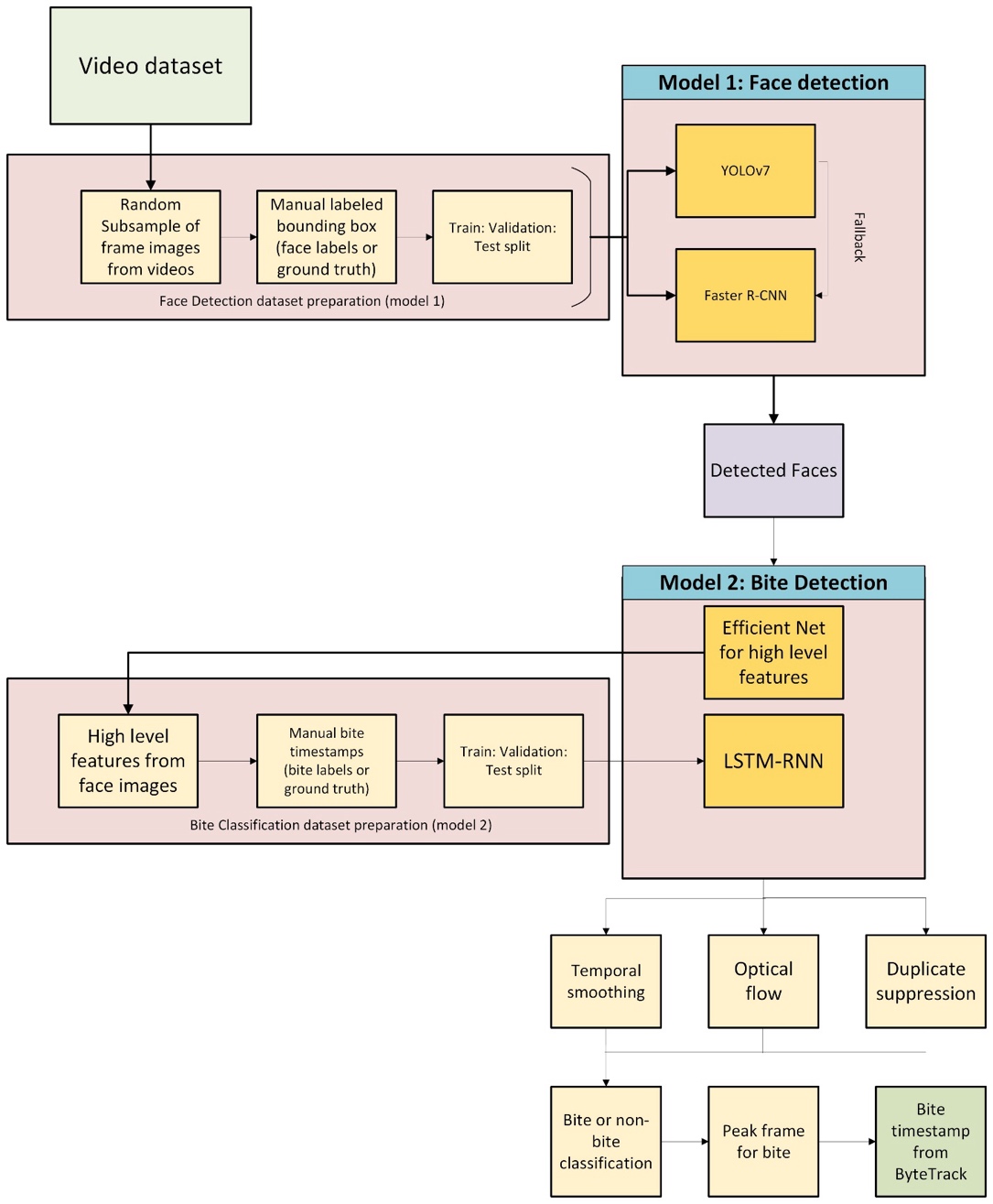


**Supplementary Figure 1:** **Detailed diagrammatic representation of ByteTrack development.** This flowchart illustrates the pipeline for face detection (Model 1) and bite classification (Model 2) from video data. The process begins with dataset preparation, where video frames are sampled and manually annotated (observational coding) with ground truth labels. For Model 1 (Face Detection), the annotated data is used to train YOLOv7 (primary) and Faster R-CNN (fallback) to detect faces. The detected faces serve as input for Model 2 (Bite Detection), where high-level features are extracted using EfficientNet and sequentially modeled with LSTM-RNN. The bite classification dataset is manually labeled with bite timestamps and split for training. Outputs undergo refinement using temporal smoothing, optical flow, and duplicate suppression, resulting in bite/non-bite classification, peak bite frames, and precise bite timestamps from ByteTrack.

##### **LOSS FUNCTION**

To handle class imbalance and encourage temporally coherent predictions, we use **CombinedTemporalFocalBCELoss**, which integrates Binary Cross-Entropy (BCE(1), Focal Loss (2), and a temporal smoothness term that discourages clusters of consecutive bite predictions (3).

**1) Binary Cross-Entropy (BCE) Loss** serves as a stable foundation for binary classification, defined as:

For logit x_t_ ∈ R, label y_t_ ∈ {0,1}, and sigmoid σ (×),

$$BCE Loss \left( x_{t}, y_{t} \right)= -\left[ y_{t}\log\sigma\left( x_{t} \right)+(1-y_{t})\log(1-\sigma(x_{t})) \right]$$

where y is the target label, x is the raw model output (logit), and σ(x) is the sigmoid function applied to the logit. This component provides a baseline loss term for model training.

2) To enhance sensitivity to the minority bite class, we incorporated **Focal Loss**. Focal Loss applies a scaling factor that emphasizes hard-to-classify examples, where it is defined as:

P_t_ is the model probability of the true class:

P_t_ = $\left\{ \begin{matrix} \sigma\left( x_{t} \right) if y_{t}=1 \\ 1- \sigma\left( x_{t} \right) if y_{t}=0 \end{matrix} \right.$

With focusing parameter γ ≥ 0 and class weight α ∈ (0,1), the focal term (per time step) is

$$FL \left( x_{t}, y_{t} \right)= - \left( 1- p_{t} \right)\log(p_{t})$$

In our experiments we set α=0.3 and γ=0.3 for the focal loss. This helps address class imbalance, particularly by giving greater weight to bite instances.

3) In addition to handling class imbalance, the loss function includes a **temporal** penalty for consecutive positive bite predictions. This component addresses the need for temporal precision by applying a penalty when consecutive bite predictions occur within a defined context window, effectively reducing false positives in close temporal proximity. When a positive prediction is followed by other positive predictions within the window, the loss is scaled by a factor “temporal_weight” = 0.8, which reduces clusters of consecutive false positives.

$$Temp \left( x \right)= \frac{1}{T} \sum_{t=1}^{T} \sum_{k=1}^{\omega} \hat{p}_{t} \hat{p}_{t}+k$$

which grows when multiple adjacent frames are predicted as positive.

4) The final **CombinedTemporalFocalBCELoss** integrates these three components into a single loss function:

*CombinedTemporalFocalBCELoss=β⋅BCE+(1−β)⋅Focal Loss+Temporal Penalty*

 where 𝛽 = 0.5, balancing contributions of BCE and Focal loss. This combined approach addresses class imbalance, improves minority-class detection, and reduces temporal overpredictions, thereby improving the model's precision in detecting individual bites.

**Supplementary Table 1: Overview of video datasets considered for training, validation, and testing.**

This table includes datasets that were discarded, along with the reasoning for exclusion, and corresponding subjects. Subject IDs are mapped to randomized IDs to ensure anonymity and prevent identification.

| Subject^ⴕ^ | Portion Size (PS) | Remarks; Train, Test, Val set | Included Or Excluded For Model Development And Testing |
| --- | --- | --- | --- |
| Sub_001 | PS1 | N/A; Subject drop-out | N/A |
|  | PS2 | Validation set | Included |
|  | PS3 | Train set | Included |
|  | PS4 | Train set | Included |
| Sub_002 | PS1 | Train set | Included |
|  | PS2 | Test set | Included |
|  | PS3 | Train set | Included |
|  | PS4 | Train set | Included |
| Sub_003 | PS1 | Train set | Included |
|  | PS2 | Test set | Included |
|  | PS3 | Train set | Included |
|  | PS4 | Train set | Included |
| Sub_004 | PS1 | Train set | Included |
|  | PS2 | Train set | Included |
|  | PS3 | Validation set | Included |
|  | PS4 | Train set | Included |
| Sub_005 | PS1 | N/A; Subject drop-out | N/A |
|  | PS2 | Validation set | Included |
|  | PS3 | Validation set | Included |
|  | PS4 | Train set | Included |
| Sub_006 | PS1 | Train set | Included |
|  | PS2 | Validation set | Included |
|  | PS3 | Test set | Included |
|  | PS4 | Validation set | Included |
| Sub_007 | PS1 | Train set | Included |
|  | PS2 | Train set | Included |
|  | PS3 | Test set | Included |
|  | PS4 | Train set | Included |
| Sub_008 | PS1 | Train set | Included |
|  | PS2 | Train set | Included |
|  | PS3 | Train set | Included |
|  | PS4 | Train set | Included |
| Sub_009 | PS1 | N/A; Not enrolled | N/A |
|  | PS2 |  |  |
|  | PS3 |  |  |
|  | PS4 |  |  |
| Sub_010 | PS1 | Test set | Included |
|  | PS2 | Train set | Included |
|  | PS3 | Train set | Included |
|  | PS4 | Test set | Included |
| Sub_011 | PS1 | Validation set | Included |
|  | PS2 | Train set | Included |
|  | PS3 | Train set | Included |
|  | PS4 | Train set | Included |
| Sub_012 | PS1 | N/A; Not enrolled | N/A |
|  | PS2 |  |  |
|  | PS3 |  |  |
|  | PS4 |  |  |
| Sub_013 | PS1 | N/A; Not enrolled | N/A |
|  | PS2 |  |  |
|  | PS3 |  |  |
|  | PS4 |  |  |
| Sub_014 | PS1 | N/A; Not enrolled | N/A |
|  | PS2 |  |  |
|  | PS3 |  |  |
|  | PS4 |  |  |
| Sub_015 | PS1 | Train set | Included |
|  | PS2 | Test set | Included |
|  | PS3 | Validation set | Included |
|  | PS4 | Face covered from head wear | Excluded |
| Sub_016 | PS1 | Train set | Included |
|  | PS2 | Train set | Included |
|  | PS3 | Face covered by hat | Excluded |
|  | PS4 | Train set | Included |
| Sub_017 | PS1 | N/A; Not enrolled | N/A |
|  | PS2 |  |  |
|  | PS3 |  |  |
|  | PS4 |  |  |
| Sub_018 | PS1 | N/A; Not enrolled | N/A |
|  | PS2 |  |  |
|  | PS3 |  |  |
|  | PS4 |  |  |
| Sub_019 | PS1 | Test set | Included |
|  | PS2 | Train set | Included |
|  | PS3 | Train set | Included |
|  | PS4 | Train set | Included |
| Sub_020 | PS1 | Train set | Included |
|  | PS2 | Train set | Included |
|  | PS3 | Validation set | Included |
|  | PS4 | Train set | Included |
| Sub_021 | PS1 | Train set | Included |
|  | PS2 | Validation set | Included |
|  | PS3 | Train set | Included |
|  | PS4 | Train set | Included |
| Sub_022 | PS1 | Train set | Included |
|  | PS2 | Train set | Included |
|  | PS3 | Train set | Included |
|  | PS4 | Validation set | Included |
| Sub_023 | PS1 | Train set | Included |
|  | PS2 | Train set | Included |
|  | PS3 | Test set | Included |
|  | PS4 | Train set | Included |
| Sub_024 | PS1 | Train set | Included |
|  | PS2 | Zoomed-in/cropped video dimensions | Excluded |
|  | PS3 | Train set | Included |
|  | PS4 | Validation set | Included |
| Sub_025 | PS1 | Validation set | Included |
|  | PS2 | Train set | Included |
|  | PS3 | Train set | Included |
|  | PS4 | Train set | Included |
| Sub_026 | PS1 | Validation set | Included |
|  | PS2 | Train set | Included |
|  | PS3 | Test set | Included |
|  | PS4 | N/A; Subject drop-out | N/A |
| Sub_027 | PS1 | Train set | Included |
|  | PS2 | Train set | Included |
|  | PS3 | Train set | Included |
|  | PS4 | Train set | Included |
| Sub_028 | PS1 | Train set | Included |
|  | PS2 | Train set | Included |
|  | PS3 | Train set | Included |
|  | PS4 | Test set | Included |
| Sub_029 | PS1 | Train set | Included |
|  | PS2 | N/A; Subject drop-out | N/A |
|  | PS3 | Train set | Included |
|  | PS4 | Train set | Included |
| Sub_030 | PS1 | Test set | Included |
|  | PS2 | Train set | Included |
|  | PS3 | Test set | Included |
|  | PS4 | Train set | Included |
| Sub_031 | PS1 | N/A; Not enrolled | N/A |
|  | PS2 |  |  |
|  | PS3 |  |  |
|  | PS4 |  |  |
| Sub_032 | PS1 | Train set | Included |
|  | PS2 | Train set | Included |
|  | PS3 | Train set | Included |
|  | PS4 | Train set | Included |
| Sub_033 | PS1 | Train set | Included |
|  | PS2 | Validation set | Included |
|  | PS3 | Train set | Included |
|  | PS4 | Train set | Included |
| Sub_034 | PS1 | Train set | Included |
|  | PS2 | Validation set | Included |
|  | PS3 | Validation set | Included |
|  | PS4 | Train set | Included |
| Sub_035 | PS1 | Train set | Included |
|  | PS2 | Train set | Included |
|  | PS3 | Train set | Included |
|  | PS4 | N/A; Subject drop-out | N/A |
| Sub_036 | PS1 | Train set | Included |
|  | PS2 | Train set | Included |
|  | PS3 | Train set | Included |
|  | PS4 | Train set | Included |
| Sub_037 | PS1 | Train set | Included |
|  | PS2 | Test set | Included |
|  | PS3 | Train set | Included |
|  | PS4 | Validation set | Included |
| Sub_038 | PS1 | Train set | Included |
|  | PS2 | Train set | Included |
|  | PS3 | Zoomed-in/cropped video dimensions | Excluded |
|  | PS4 | Missing ground truth files | Excluded |
| Sub_039 | PS1 | Train set | Included |
|  | PS2 | Train set | Included |
|  | PS3 | Train set | Included |
|  | PS4 | Train set | Included |
| Sub_040 | PS1 | N/A; Not enrolled | N/A |
|  | PS2 |  |  |
|  | PS3 |  |  |
|  | PS4 |  |  |
| Sub_041 | PS1 | Train set | Included |
|  | PS2 | Different camera angle | Excluded |
|  | PS3 | Train set | Included |
|  | PS4 | Train set | Included |
| Sub_042 | PS1 | Validation set | Included |
|  | PS2 | Train set | Included |
|  | PS3 | Train set | Included |
|  | PS4 | Train set | Included |
| Sub_043 | PS1 | N/A; Not enrolled | N/A |
|  | PS2 |  |  |
|  | PS3 |  |  |
|  | PS4 |  |  |
| Sub_044 | PS1 | Train set | Included |
|  | PS2 | Train set | Included |
|  | PS3 | Train set | Included |
|  | PS4 | Test set | Included |
| Sub_045 | PS1 | N/A; Not enrolled | N/A |
|  | PS2 |  |  |
|  | PS3 |  |  |
|  | PS4 |  |  |
| Sub_046 | PS1 | N/A; Not enrolled | N/A |
|  | PS2 |  |  |
|  | PS3 |  |  |
|  | PS4 |  |  |
| Sub_047 | PS1 | Validation set | Included |
|  | PS2 | Train set | Included |
|  | PS3 | Train set | Included |
|  | PS4 | Train set | Included |
| Sub_048 | PS1 | N/A; Not enrolled | N/A |
|  | PS2 |  |  |
|  | PS3 |  |  |
|  | PS4 |  |  |
| Sub_049 | PS1 | Train set | Included |
|  | PS2 | Train set | Included |
|  | PS3 | Test set | Included |
|  | PS4 | Train set | Included |
| Sub_050 | PS1 | N/A; Not enrolled | N/A |
|  | PS2 |  |  |
|  | PS3 |  |  |
|  | PS1 |  |  |
| Sub_051 | PS1 | Validation set | Included |
|  | PS2 | Validation set | Included |
|  | PS3 | Test set | Included |
|  | PS4 | Train set | Included |
| Sub_052 | PS1 | Train set | Included |
|  | PS2 | Train set | Included |
|  | PS3 | Train set | Included |
|  | PS4 | Train set | Included |
| Sub_053 | PS1 | Train set | Included |
|  | PS2 | Train set | Included |
|  | PS3 | Test set | Included |
|  | PS4 | Train set | Included |
| Sub_054 | PS1 | N/A; Not enrolled | N/A |
|  | PS2 |  |  |
|  | PS3 |  |  |
|  | PS4 |  |  |
| Sub_055 | PS1 | Train set | Included |
|  | PS2 | Validation set | Included |
|  | PS3 | Train set | Included |
|  | PS4 | N/A; Subject drop-out | N/A |
| Sub_056 | PS1 | Validation set | Included |
|  | PS2 | Train set | Included |
|  | PS3 | Train set | Included |
|  | PS4 | Train set | Included |
| Sub_057 | PS1 | N/A; Not enrolled |  |
|  | PS2 |  |  |
|  | PS3 |  |  |
|  | PS4 |  |  |
| Sub_058 | PS1 | Train set | Included |
|  | PS2 | Train set | Included |
|  | PS3 | Train set | Included |
|  | PS4 | Train set | Included |
| Sub_059 | PS1 | Train set | Included |
|  | PS2 | Train set | Included |
|  | PS3 | Train set | Included |
|  | PS4 | Validation set | Included |
| Sub_060 | PS1 | N/A; Not enrolled | N/A |
|  | PS2 |  |  |
|  | PS3 |  |  |
|  | PS4 |  |  |
| Sub_061 | PS1 | N/A; Not enrolled | N/A |
|  | PS2 |  |  |
|  | PS3 |  |  |
|  | PS4 |  |  |
| Sub_062 | PS1 | Train set | Included |
|  | PS2 | Test set | Included |
|  | PS3 | Train set | Included |
|  | PS4 | Validation set | Included |
| Sub_063 | PS1 | N/A; Not enrolled | N/A |
|  | PS2 |  |  |
|  | PS3 |  |  |
|  | PS4 |  |  |
| Sub_064 | PS1 | N/A; Not enrolled | N/A |
|  | PS2 |  |  |
|  | PS3 |  |  |
|  | PS4 |  |  |
| Sub_065 | PS1 | N/A; Not enrolled | N/A |
|  | PS2 |  |  |
|  | PS3 |  |  |
|  | PS4 |  |  |
| Sub_066 | PS1 | N/A; Not enrolled | N/A |
|  | PS2 |  |  |
|  | PS3 |  |  |
|  | PS4 |  |  |
| Sub_067 | PS1 | Train set | Included |
|  | PS2 | Train set | Included |
|  | PS3 | Train set | Included |
|  | PS4 | Train set | Included |
| Sub_068 | PS1 | Train set | Included |
|  | PS2 | Validation set | Included |
|  | PS3 | Train set | Included |
|  | PS4 | Train set | Included |
| Sub_069 | PS1 | N/A; Not enrolled | N/A |
|  | PS2 |  |  |
|  | PS3 |  |  |
|  | PS4 |  |  |
| Sub_070 | PS1 | N/A; Subject drop-out | N/A |
|  | PS2 |  |  |
|  | PS3 | Train set | Included |
|  | PS4 | N/A; Subject drop-out | N/A |
| Sub_071 | PS1 | N/A; Not enrolled | N/A |
|  | PS2 |  |  |
|  | PS3 |  |  |
|  | PS4 |  |  |
| Sub_072 | PS1 | Train set | Included |
|  | PS2 | Train set | Included |
|  | PS3 | Train set | Included |
|  | PS4 | Train set | Included |
| Sub_073 | PS1 | Train set | Included |
|  | PS2 | Train set | Included |
|  | PS3 | Train set | Included |
|  | PS4 | Train set | Included |
| Sub_074 | PS1 | Validation set | Included |
|  | PS2 | Train set | Included |
|  | PS3 | Child not facing camera | Excluded |
|  | PS4 | Validation set | Included |
| Sub_075 | PS1 | Train set | Included |
|  | PS2 | Validation set | Included |
|  | PS3 | Train set | Included |
|  | PS4 | Train set | Included |
| Sub_076 | PS1 | Train set | Included |
|  | PS2 | Train set | Included |
|  | PS3 | Child not facing camera | Excluded |
|  | PS4 | Test set | Included |
| Sub_077 | PS1 | N/A; Not enrolled | N/A |
|  | PS2 |  |  |
|  | PS3 |  |  |
|  | PS4 |  |  |
| Sub_078 | PS1 | N/A; Not enrolled | N/A |
|  | PS2 |  |  |
|  | PS3 |  |  |
|  | PS4 |  |  |
| Sub_079 | PS1 | Train set | Included |
|  | PS2 | Train set | Included |
|  | PS3 | Train set | Included |
|  | PS4 | N/A; Subject drop-out | N/A |
| Sub_080 | PS1 | N/A; Not enrolled | N/A |
|  | PS2 |  |  |
|  | PS3 |  |  |
|  | PS4 |  |  |
| Sub_081 | PS1 | Validation set | Included |
|  | PS2 | Train set | Included |
|  | PS3 | Train set | Included |
|  | PS4 | Train set | Included |
| Sub_082 | PS1 | Validation set | Included |
|  | PS2 | Train set | Included |
|  | PS3 | Train set | Included |
|  | PS4 | Validation set | Included |
| Sub_083 | PS1 | Train set | Included |
|  | PS2 | Train set | Included |
|  | PS3 | Validation set | Included |
|  | PS4 | Validation set | Included |
| Sub_084 | PS1 | Train set | Included |
|  | PS2 | Train set | Included |
|  | PS3 | Train set | Included |
|  | PS4 | Test set | Included |
| Sub_085 | PS1 | Train set | Included |
|  | PS2 | Validation set | Included |
|  | PS3 | Test set | Included |
|  | PS4 | Train set | Included |
| Sub_086 | PS1 | Test set | Included |
|  | PS2 | Train set | Included |
|  | PS3 | Train set | Included |
|  | PS4 | Train set | Included |
| Sub_087 | PS1 | N/A; Not enrolled | N/A |
|  | PS2 |  |  |
|  | PS3 |  |  |
|  | PS4 |  |  |
| Sub_088 | PS1 | Train set | Included |
|  | PS2 | Train set | Included |
|  | PS3 | Train set | Included |
|  | PS4 | Test set | Included |
| Sub_089 | PS1 | Train set | Included |
|  | PS2 | Train set | Included |
|  | PS3 | Test set | Included |
|  | PS4 | Train set | Included |
| Sub_090 | PS1 | N/A; Subject drop-out | N/A |
|  | PS2 |  |  |
|  | PS3 | Train set | Included |
|  | PS4 | N/A; Subject drop-out |  |
| Sub_091 | PS1 | N/A; Not enrolled | N/A |
|  | PS2 |  |  |
|  | PS3 |  |  |
|  | PS4 |  |  |
| Sub_092 | PS1 | N/A; Subject drop-out | N/A |
|  | PS2 |  |  |
|  | PS3 |  |  |
|  | PS4 | Missing ground truth files | Excluded |
| Sub_093 | PS1 | N/A; Not enrolled | N/A |
|  | PS2 |  |  |
|  | PS3 |  |  |
|  | PS4 |  |  |
| Sub_094 | PS1 | Train set | Included |
|  | PS2 | Train set | Included |
|  | PS3 | Train set | Included |
|  | PS4 | Train set | Included |
| Sub_095 | PS1 | Validation set | Included |
|  | PS2 | Test set | Included |
|  | PS3 | Train set | Included |
|  | PS4 | Train set | Included |
| Sub_096 | PS1 | Train set | Included |
|  | PS2 | Validation set | Included |
|  | PS3 | Train set | Included |
|  | PS4 | Missing ground truth files | Excluded |
| Sub_097 | PS1 | Train set | Included |
|  | PS2 | Test set | Included |
|  | PS3 | Train set | Included |
|  | PS4 | Test set | Included |
| Sub_098 | PS1 | Train set | Included |
|  | PS2 | Validation set | Included |
|  | PS3 | Train set | Included |
|  | PS4 | Test set | Included |
| Sub_099 | PS1 | N/A; Not enrolled | N/A |
|  | PS2 |  |  |
|  | PS3 |  |  |
|  | PS4 |  |  |
| Sub_100 | PS1 | Test set | Included |
|  | PS2 | Train set | Included |
|  | PS3 | Test set | Included |
|  | PS4 | Train set | Included |
| Sub_101 | PS1 | N/A; Not enrolled | N/A |
|  | PS2 |  |  |
|  | PS3 |  |  |
|  | PS4 |  |  |
| Sub_102 | PS1 | Train set | Included |
|  | PS2 | Train set | Included |
|  | PS3 | Test set | Included |
|  | PS4 | Train set | Included |
| Sub_103 | PS1 | Train set | Included |
|  | PS2 | Train set | Included |
|  | PS3 | Test set | Included |
|  | PS4 | Validation set | Included |
| Sub_104 | PS1 | Train set | Included |
|  | PS2 | Test set | Included |
|  | PS3 | Test set | Included |
|  | PS4 | Train set | Included |
| Sub_105 | PS1 | Train set | Included |
|  | PS2 | Train set | Included |
|  | PS3 | Video recording error (empty video recording) | Excluded |
|  | PS4 | Validation set | Included |
| Sub_106 | PS1 | Train set | Included |
|  | PS2 | N/A; Subject drop-out | N/A |
|  | PS3 |  |  |
|  | PS4 | Train set | Included |
| Sub_107 | PS1 | Train set | Included |
|  | PS2 | Train set | Included |
|  | PS3 | Train set | Included |
|  | PS4 | Validation set | Included |
| Sub_108 | PS1 | Train set | Included |
|  | PS2 | Train set | Included |
|  | PS3 | Train set | Included |
|  | PS4 | Train set | Included |
| Sub_109 | PS1 | N/A; Not enrolled | N/A |
|  | PS2 |  |  |
|  | PS3 |  |  |
|  | PS4 |  |  |
| Sub_110 | PS1 | N/A; Not enrolled | N/A |
|  | PS2 |  |  |
|  | PS3 |  |  |
|  | PS4 |  |  |
| Sub_111 | PS1 | N/A; Not enrolled | N/A |
|  | PS2 |  |  |
|  | PS3 |  |  |
|  | PS4 |  |  |
| Sub_112 | PS1 | Train set | Included |
|  | PS2 | Test set | Included |
|  | PS3 | Train set | Included |
|  | PS4 | Train set | Included |
| Sub_113 | PS1 | N/A; Not enrolled | N/A |
|  | PS2 |  |  |
|  | PS3 |  |  |
|  | PS4 |  |  |
| Sub_114 | PS1 | Train set | Included |
|  | PS2 | Validation set | Included |
|  | PS3 | Train set | Included |
|  | PS4 | Train set | Included |
| Sub_115 | PS1 | Train set | Included |
|  | PS2 | Train set | Included |
|  | PS3 | N/A; Subject drop-out | N/A |
|  | PS4 |  |  |
| Sub_116 | PS1 | Train set | Included |
|  | PS2 | Validation set | Included |
|  | PS3 | Train set | Included |
|  | PS4 | Test set | Included |
| Sub_117 | PS1 | Validation set | Included |
|  | PS2 | Train set | Included |
|  | PS3 | Train set | Included |
|  | PS4 | Train set | Included |
| Sub_118 | PS1 | Train set | Included |
|  | PS2 | Train set | Included |
|  | PS3 | Test set | Included |
|  | PS4 | Train set | Included |
| Sub_119 | PS1 | Validation set | Included |
|  | PS2 | Test set | Included |
|  | PS3 | Train set | Included |
|  | PS4 | Train set | Included |
| Sub_120 | PS1 | Test set | Included |
|  | PS2 | Validation set | Included |
|  | PS3 | Train set | Included |
|  | PS4 | N/A; Subject drop-out | N/A |
| Sub_121 | PS1 | N/A; Not enrolled | N/A |
|  | PS2 |  |  |
|  | PS3 |  |  |
|  | PS4 |  |  |
| Sub_122 | PS1 | Test set | Included |
|  | PS2 | Test set | Included |
|  | PS3 | Train set | Included |
|  | PS4 | Test set | Included |
| Sub_123 | PS1 | N/A; Not enrolled | N/A |
|  | PS2 |  |  |
|  | PS3 |  |  |
|  | PS4 |  |  |
| Sub_124 | PS1 | Validation set | Included |
|  | PS2 | Train set | Included |
|  | PS3 | Test set | Included |
|  | PS4 | Test set | Included |
| Sub_125 | PS1 | Validation set | Included |
|  | PS2 | Train set | Included |
|  | PS3 | Train set | Included |
|  | PS4 | Train set | Included |
| Sub_126 | PS1 | N/A; Not enrolled | N/A |
|  | PS2 |  |  |
|  | PS3 |  |  |
|  | PS4 |  |  |
| Sub_127 | PS1 | Train set | Included |
|  | PS2 | Train set | Included |
|  | PS3 | Train set | Included |
|  | PS4 | Validation set | Included |
| Sub_128 | PS1 | Train set | Included |
|  | PS2 | Train set | Included |
|  | PS3 | Train set | Included |
|  | PS4 | Corrupt video file | Excluded |
| Sub_129 | PS1 | Train set | Included |
|  | PS2 | Train set | Included |
|  | PS3 | Train set | Included |
|  | PS4 | Test set | Included |
| Sub_130 | PS1 | N/A; Not enrolled | N/A |
|  | PS2 |  |  |
|  | PS3 |  |  |
|  | PS4 |  |  |
| Sub_131 | PS1 | Train set | Included |
|  | PS2 | Train set | Included |
|  | PS3 | Train set | Included |
|  | PS4 | Train set | Included |
| Sub_132 | PS1 | Train set | Included |
|  | PS2 | N/A; Subject drop-out | N/A |
|  | PS3 | Test set | Included |
|  | PS4 | Train set | Included |
| Sub_133 | PS1 | Test set | Included |
|  | PS2 | Validation set | Included |
|  | PS3 | N/A; Subject drop-out | N/A |
|  | PS4 |  |  |

^ⴕ^ Subject IDs are mapped to randomized IDs to ensure anonymity and prevent identification.

**Supplementary Table 2: Model Performance on test set videos (n=51)**

| **Subject^ⴕ^** | **Visit (V), Portion Size (PS)** | **True Positives (TP)** | **False Positives (FP)** | **False Negatives (FN)** | **Precision (P)** | **Recall ®** | **F1 Score** | **Manual Bite Count (Ground truth)** | **Modeled Bite Count (Predicted bite count)** | **Modeled Meal Duration (min)** | **Manual Meal Duration (min)** | **Manual Bite Rate (bites/min)** | **Modeled Bite Rate (bites/min)** |
| --- | --- | --- | --- | --- | --- | --- | --- | --- | --- | --- | --- | --- | --- |
| Sub_002 | V2, PS2 | 68 | 11 | 12 | 0.86 | 0.85 | 0.86 | 39 | 79 | 7.59 | 10.17 | 3.84 | 10.4 |
| Sub_003 | V3, PS2 | 46 | 18 | 27 | 0.72 | 0.63 | 0.67 | 41 | 64 | 7.21 | 9.97 | 4.11 | 8.87 |
| Sub_006 | V2, PS3 | 23 | 19 | 46 | 0.55 | 0.33 | 0.41 | 65 | 42 | 13 | 6.08 | 10.7 | 3.23 |
| Sub_007 | V5, PS3 | 243 | 106 | 20 | 0.7 | 0.92 | 0.8 | 67 | 349 | 18 | 24.25 | 2.76 | 19.39 |
| Sub_010 | V2, PS1 | 77 | 11 | 18 | 0.88 | 0.81 | 0.84 | 48 | 88 | 5.92 | 7.91 | 6.07 | 14.86 |
| Sub_010 | V3, PS4 | 252 | 6 | 37 | 0.98 | 0.87 | 0.92 | 90 | 258 | 9.79 | 14.05 | 6.4 | 26.36 |
| Sub_015 | V2, PS2 | 80 | 4 | 72 | 0.95 | 0.53 | 0.68 | 134 | 84 | 13.03 | 15.11 | 8.87 | 6.45 |
| Sub_019 | V3, PS1 | 17 | 0 | 15 | 1 | 0.53 | 0.69 | 27 | 17 | 2.92 | 4.023 | 6.72 | 5.82 |
| Sub_023 | V2, PS3 | 51 | 0 | 44 | 1 | 0.54 | 0.7 | 65 | 51 | 5.32 | 10.4 | 6.25 | 9.58 |
| Sub_026 | V5, PS3 | 128 | 5 | 34 | 0.96 | 0.79 | 0.87 | 79 | 133 | 9.62 | 14.95 | 5.29 | 13.82 |
| Sub_028 | V3, PS4 | 32 | 13 | 20 | 0.71 | 0.62 | 0.66 | 42 | 45 | 16.88 | 17.59 | 2.38 | 2.67 |
| Sub_030 | V3, PS3 | 137 | 17 | 29 | 0.89 | 0.83 | 0.86 | 74 | 154 | 9.63 | 15.53 | 4.76 | 15.98 |
| Sub_030 | V5, PS1 | 135 | 8 | 18 | 0.94 | 0.88 | 0.91 | 51 | 143 | 6.93 | 11.24 | 4.54 | 20.63 |
| Sub_037 | V4, PS2 | 55 | 6 | 31 | 0.9 | 0.64 | 0.75 | 64 | 61 | 10.45 | 11.82 | 5.41 | 5.84 |
| Sub_044 | V3, PS4 | 45 | 11 | 28 | 0.8 | 0.62 | 0.7 | 56 | 56 | 12.33 | 13.34 | 4.2 | 4.54 |
| Sub_047 | V4, PS2 | 17 | 9 | 28 | 0.65 | 0.38 | 0.48 | 44 | 26 | 10.4 | 8.35 | 5.27 | 2.51 |
| Sub_049 | V3, PS3 | 46 | 0 | 31 | 1 | 0.6 | 0.75 | 62 | 46 | 10.39 | 12.81 | 4.84 | 4.43 |
| Sub_051 | V5, PS3 | 52 | 1 | 45 | 0.98 | 0.54 | 0.69 | 78 | 53 | 8.5 | 11.08 | 7.72 | 6.24 |
| Sub_053 | V3, PS3 | 123 | 33 | 96 | 0.79 | 0.56 | 0.66 | 159 | 156 | 23.16 | 31.88 | 4.99 | 6.74 |
| Sub_054 | V4, PS2 | 83 | 9 | 16 | 0.9 | 0.84 | 0.87 | 55 | 92 | 8.79 | 10.62 | 5.18 | 10.46 |
| Sub_062 | V5, PS2 | 105 | 136 | 29 | 0.44 | 0.78 | 0.56 | 74 | 241 | 22.06 | 26.92 | 2.75 | 10.93 |
| Sub_075 | V5, PS3 | 144 | 7 | 117 | 0.95 | 0.55 | 0.7 | 181 | 151 | 8.27 | 13.36 | 13.55 | 18.26 |
| Sub_076 | V3, PS4 | 275 | 79 | 20 | 0.78 | 0.93 | 0.85 | 116 | 354 | 23.9 | 28.6 | 4.06 | 14.81 |
| Sub_084 | V4, PS4 | 77 | 118 | 18 | 0.4 | 0.81 | 0.53 | 44 | 195 | 19.15 | 20.73 | 2.12 | 10.19 |
| Sub_085 | V4, PS3 | 53 | 4 | 58 | 0.93 | 0.48 | 0.63 | 92 | 57 | 10.89 | 15.51 | 5.93 | 5.23 |
| Sub_086 | V5, PS1 | 140 | 8 | 37 | 0.95 | 0.79 | 0.86 | 96 | 148 | 10.17 | 13.93 | 6.89 | 14.55 |
| Sub_088 | V3, PS4 | 160 | 6 | 52 | 0.96 | 0.75 | 0.85 | 122 | 166 | 14.66 | 22.07 | 5.53 | 11.33 |
| Sub_089 | V3, PS3 | 172 | 23 | 65 | 0.88 | 0.73 | 0.8 | 147 | 195 | 18.84 | 27.11 | 5.43 | 10.36 |
| Sub_095 | V4, PS2 | 14 | 11 | 85 | 0.56 | 0.14 | 0.23 | 102 | 25 | 15.63 | 18.37 | 5.55 | 1.6 |
| Sub_097 | V2, PS4 | 50 | 70 | 8 | 0.42 | 0.86 | 0.56 | 37 | 120 | 19.91 | 14.61 | 2.53 | 6.03 |
| Sub_097 | V4, PS2 | 38 | 9 | 3 | 0.81 | 0.93 | 0.86 | 22 | 47 | 6.97 | 6.95 | 3.17 | 6.75 |
| Sub_098 | V2, PS4 | 185 | 5 | 33 | 0.97 | 0.85 | 0.91 | 82 | 190 | 7.58 | 13.35 | 6.14 | 25.06 |
| Sub_100 | V2, PS1 | 13 | 21 | 52 | 0.38 | 0.2 | 0.26 | 66 | 34 | 23.19 | 11.28 | 5.85 | 1.47 |
| Sub_100 | V5, PS3 | 30 | 4 | 78 | 0.88 | 0.28 | 0.42 | 108 | 34 | 22.36 | 26.07 | 4.14 | 1.52 |
| Sub_102 | V5, PS3 | 41 | 13 | 12 | 0.76 | 0.77 | 0.77 | 29 | 54 | 6.65 | 8.26 | 3.51 | 8.12 |
| Sub_103 | V4, PS3 | 47 | 30 | 27 | 0.61 | 0.64 | 0.62 | 59 | 77 | 19.37 | 27.25 | 2.17 | 3.97 |
| Sub_104 | V4, PS2 | 48 | 44 | 53 | 0.52 | 0.48 | 0.5 | 92 | 92 | 20.31 | 29.89 | 3.08 | 4.53 |
| Sub_104 | V5, PS3 | 191 | 93 | 13 | 0.67 | 0.94 | 0.78 | 71 | 284 | 17.17 | 29.86 | 2.38 | 16.54 |
| Sub_112 | V2, PS2 | 260 | 212 | 16 | 0.55 | 0.94 | 0.7 | 74 | 472 | 19.99 | 15.13 | 4.89 | 23.61 |
| Sub_116 | V3, PS4 | 113 | 80 | 24 | 0.59 | 0.83 | 0.69 | 80 | 193 | 22.18 | 27.1509 | 2.95 | 8.7 |
| Sub_118 | V5, PS3 | 85 | 18 | 14 | 0.83 | 0.86 | 0.84 | 43 | 103 | 10.81 | 14.7 | 2.92 | 9.54 |
| Sub_119 | V5, PS2 | 664 | 49 | 74 | 0.93 | 0.9 | 0.92 | 172 | 713 | 14.65 | 28.82 | 5.96 | 48.67 |
| Sub_122 | V2, PS2 | 51 | 6 | 71 | 0.89 | 0.42 | 0.57 | 115 | 57 | 10.5 | 12.08 | 9.52 | 5.43 |
| Sub_122 | V4, PS4 | 66 | 4 | 65 | 0.94 | 0.5 | 0.66 | 108 | 70 | 8.89 | 12.08 | 8.94 | 7.88 |
| Sub_122 | V5, PS1 | 53 | 6 | 36 | 0.9 | 0.6 | 0.72 | 73 | 59 | 7.61 | 9.054 | 8.06 | 7.76 |
| Sub_124 | V2, PS3 | 125 | 85 | 19 | 0.6 | 0.87 | 0.71 | 46 | 210 | 13.37 | 19.19 | 2.4 | 15.71 |
| Sub_124 | V3, PS4 | 45 | 41 | 11 | 0.52 | 0.8 | 0.64 | 29 | 86 | 8.62 | 10.97 | 2.65 | 9.97 |
| Sub_129 | V3, PS4 | 137 | 76 | 39 | 0.64 | 0.78 | 0.7 | 94 | 213 | 18.7 | 28.42 | 3.3 | 11.39 |
| Sub_132 | V5, PS3 | 54 | 11 | 34 | 0.83 | 0.61 | 0.71 | 65 | 65 | 11.39 | 14.29 | 4.54 | 5.7 |
| Sub_133 | V2, PS4 | 110 | 0 | 12 | 1 | 0.9 | 0.95 | 36 | 110 | 3.024 | 4.27 | 8.42 | 36.37 |
| Sub_133 | V4, PS1 | 46 | 8 | 4 | 0.85 | 0.92 | 0.89 | 16 | 54 | 2.42 | 2.74 | 5.84 | 22.39 |

^ⴕ^Subject IDs are mapped to randomized IDs to ensure anonymity and prevent identification.


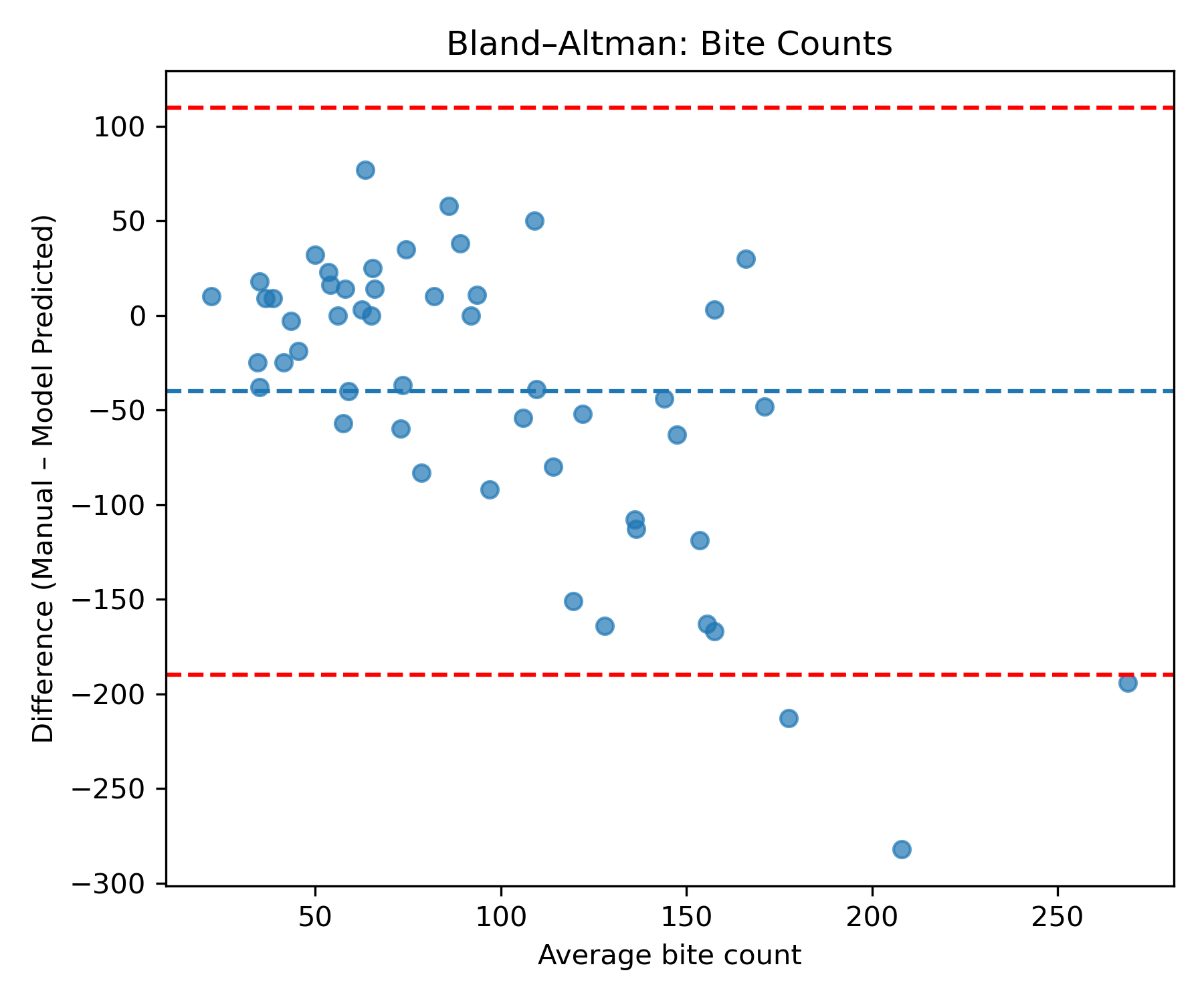


**Supplementary Figure 2:** Bland-Altman plot showing differences between bite counts between manually annotated bite count and model predicted bite count across test set (n=51 videos)

**REFERENCES**

1. Goodfellow I, Bengio Y, Courville A. Deep learning. Cambridge, Massachusetts: The MIT Press; 2016. 775 p. (Adaptive computation and machine learning).

2. Lin TY, Goyal P, Girshick R, He K, Dollár P. Focal Loss for Dense Object Detection. In: 2017 IEEE International Conference on Computer Vision (ICCV). 2017. p. 2999–3007. https://doi.org/10.1109/ICCV.2017.324

3. Szegedy C, Vanhoucke V, Ioffe S, Shlens J, Wojna Z. Rethinking the Inception Architecture for Computer Vision. 2016 IEEE Conference on Computer Vision and Pattern Recognition (CVPR). 2016 Jun;2818–26. https://doi.org/10.1109/CVPR.2016.308
